# Influence of Microstructure on the Elution Behavior of Gradient Copolymers in Different Modes of Liquid Interaction Chromatography

**DOI:** 10.1021/acs.analchem.2c00193

**Published:** 2022-05-23

**Authors:** Blaž Zdovc, Heng Li, Junpeng Zhao, David Pahovnik, Ema Žagar

**Affiliations:** † Department of Polymer Chemistry and Technology, National Institute of Chemistry, Hajdrihova 19, Ljubljana SI-1000, Slovenia; ‡ Faculty of Materials Science and Engineering, South China University of Technology, 381 Wushan Road, Guangzhou 510641, P. R. China

## Abstract

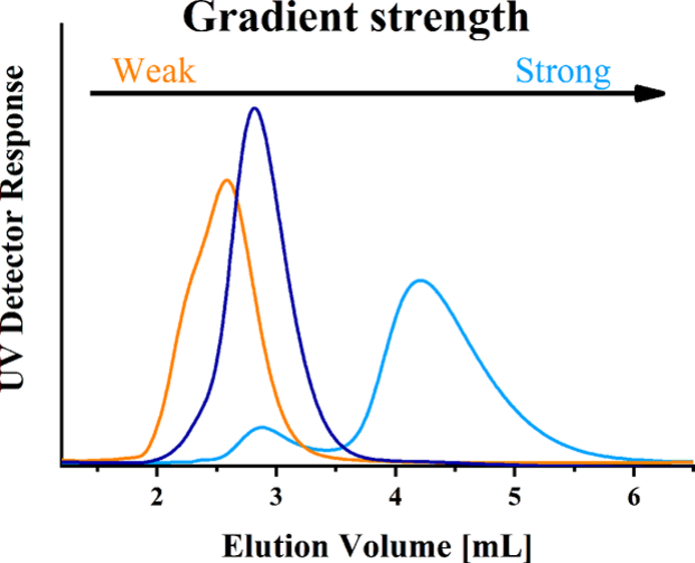

We studied the influence of microstructure on the chromatographic behavior of gradient copolymers with different gradient strengths and block copolymer with completely segregated blocks by using gradient liquid adsorption chromatography (gLAC) and liquid chromatography at critical conditions (LCCC) for one of the copolymer constituents. The copolymers consist of repeating units of poly(propylene oxide) and poly(propylene phthalate) and have comparable average chemical composition and molar mass, and a narrow molar mass distribution to avoid as much as possible the influence of these parameters on the elution behavior of the copolymers. On both reversed stationary phases, the elution volume of gradient copolymers increases with the increasing strength of the gradient. The results indicate that for both modes of liquid interaction chromatography, it is important to consider the effect of microstructure on the elution behavior of the gradient copolymers in addition to the copolymer chemical composition and molar mass in the case of gLAC and the length of the chromatographically visible copolymer constituent in the case of LCCC.

## Introduction

(Co)polymers can exhibit heterogeneity in several properties simultaneously, for example, molar mass, chemical composition, functionality, architecture, and microstructure.^1,2^ These properties and their distributions affect the physicochemical properties of (co)polymers.^1^ Therefore, the accurate characterization of these parameters is crucial to understanding the relationship between the structure and properties of complex polymers.^1,3^ The above-mentioned properties and their distributions are usually determined by various liquid separation techniques; that is, molar mass distribution by size-exclusion chromatography (SEC), chemical composition distribution by liquid adsorption chromatography (LAC),^4−10^ while end-group functionality and block length distributions in block copolymers are determined by liquid chromatography under critical conditions (LCCC).^11−14^ LAC and LCCC are also known to be useful techniques for determining various types of microstructures, such as tacticity in polymethacrylates,^15−17^ and various arrangements of substituents at an olefinic double bond resulting from different polymerization patterns of dienes.^18−21^ Molar mass, chemical composition, and comonomer distributions can also be characterized by temperature gradient interaction chromatography.^22−25^ Because complex polymers exhibit heterogeneity in multiple properties simultaneously, their characterization by a one-dimensional (1D) liquid chromatographic technique is insufficient and instead requires the coupling of various 1D liquid chromatographic techniques into a two-dimensional LC (2D-LC) system that allows the simultaneous characterization of two heterogeneous (co)polymer properties.^1^

Depending on the distribution of two different comonomer units along the copolymer chain, the main types of copolymers are random, alternating, gradient, and block copolymers. Simple gradient copolymers exhibit a gradual transition in composition from predominantly A to predominantly B comonomer repeating units along the copolymer chains.^26,27^ This transition is defined by the strength of the gradient, which affects the properties of gradient copolymers in the solution and in the solid state. In gradient copolymers with a strong gradient, the transition from A to B repeating units is rapid, and such gradient copolymers exhibit properties similar to those of analogous block copolymers. Gradient copolymers exhibit a broad glass transition temperature due to the incomplete segregation of microphases, which makes them excellent damping materials.^26−31^ Amphiphilic gradient copolymers, similar to block copolymers, exhibit the ability to self-assemble into supramolecular structures depending on the composition of the solvent,^32−35^ the pH of the environment,^36−38^ or the temperature,^39−44^ which has been exploited in various biomedical applications.^26,28^ Previous studies characterizing gradient copolymers with different gradient profiles were limited to determining the glass transition region by dynamic mechanical analysis^27^ and differential scanning calorimetry (DSC),^30^ the extent of aggregation by turbidimetric titrations,^31^ and the degree of phase separation by theoretical studies^45,46^ and dynamic light scattering.^47,48^ Recently, gradient copolymers have gained attention as cost-effective alternatives to block copolymers in areas such as thermoplastic elastomers, compatibilizers for immiscible polymer blends, or as stabilizers for emulsions and dispersions.^26,28,29^ At the same chemical composition, a stronger gradient facilitates the phase separation of gradient copolymers, as evident from the lower product of the degree of polymerization of the polymer chain (*N*) and the effective Flory–Huggins interaction parameter, χ, at the critical transition point from order to disorder.^49^ The effect of the gradient profile on self-assembly behavior and interfacial properties has been studied theoretically^50,51^ and by examining the midpoint and the width of the glass transition.^52^

The effect of the sequence distribution of comonomer units in alternating, statistical, and block copolymers on elution behavior in gradient LAC (gLAC) was considered in a theoretical study by Brun.^53^ In the derived expressions, the effective interaction energy of alternating, statistical, and block copolymers with a chromatographic stationary phase depends on chemical composition and microstructure, while the effect of molar mass on elution behavior^54^ was neglected by assuming identical molar masses for all types of copolymers.^53^ Considering the equimolar chemical composition of the copolymers, the results of the derived expressions showed that the effective interaction energy increases in order alternating, statistical to the block copolymer, so that the order of elution from the column should be the same. The results of this study are consistent with the results of Monte Carlo computer simulations for the partition coefficient of statistical copolymers with different degrees of blockiness.^55^ In addition, the results of computer simulations using the Kremer–Grest bead–spring model show higher dynamic modulus values as a function of distance from the nanoparticle surface for the block copolymer than for the alternating copolymer, indicating a higher interaction energy of the block copolymer with the nanoparticle surface.^56^ The results of the theoretical studies were consistent with the results of experimental studies by Brun^57^ and Peltier,^58^ who studied the elution behavior of random and block copolymers with comparable chemical composition and molar mass, where the late elution of the block copolymer compared to the random copolymer was explained by the different microstructures. In contrast, Augenstein and Müller^59^ attributed the late elution of block copolymers compared to random copolymers to the formation of supramolecular micelles. Separation by chemical composition and microstructure in terms of ethylene or propylene sequence lengths has also been successfully performed for ethylene-*co*-propylene random copolymers using high-temperature solvent gradient interaction chromatography.^60^

In this work, we studied the copolymers consisting of A (poly(propylene phthalate); P(POPA)) and B (poly(propylene oxide); PPO) repeating units that are differently distributed along the copolymer chains and thus have different microstructure; that is, block and three gradient copolymers with different gradient profiles. The influence of microstructure on the elution behavior of copolymers was investigated in different modes of liquid interaction chromatography, that is, gLAC and LCCC, where separation is controlled by enthalpic interactions.

## Experimental Section

Solvents for LC measurements: chloroform (CHCl_3_, ≥99.8%), acetonitrile (ACN, ≥99.9%), and tetrahydrofuran (THF) (Riedel de Haën, Germany, ≥99.9%) were used as received.

### ^**1**^H NMR Spectroscopy

The ^1^H nuclear magnetic resonance (NMR) spectra of the homo- and copolymers and their fractions were recorded in CDCl_3_ using a 600 MHz Neo Avance spectrometer (Bruker; USA). Tetramethylsilane (Me_4_Si, δ = 0 ppm) was used as an internal chemical shift standard.

### SEC with a Multidetection System Consisting of Ultraviolet, Multiangle Light Scattering, and Differential Refractive Index Detectors (SEC/UV-MALS-RI)

The molar mass characteristics of the homo- and copolymers were determined by SEC coupled with a multidetection system consisting of an ultraviolet (UV) detector operating at a wavelength of 283 nm (Agilent 1260 DAD VL, Agilent Technologies, USA), a multiangle light scattering (MALS) photometer with 18 angles (DAWN HELEOS-II, Wyatt Technology Corporation, USA), and a refractive index (RI) detector (Optilab T-rEX, Wyatt Technology Corporation, USA). The SEC/UV-MALS-RI allows determination of the molar masses of the copolymers and the molar masses of the individual copolymer constituents and thus the copolymer chemical composition (Supporting Information; Calculation Procedure in SEC/UV-MALS-RI), if the copolymers have a narrow distribution in chemical composition and the two copolymer constituents give different ratios of UV to RI signals. In our case, only the P(POPA) constituent was UV active at 283 nm, while the PPO is invisible at this wavelength. The input parameters required for such a calculation are the specific RI increments (d*n*/d*c*) and the extinction coefficients (ε) of the two copolymer constituents, which were determined for the corresponding homopolymers assuming 100% mass recovery of the samples from the column (Table S1). Separations of the (co)polymers were performed at room temperature in CHCl_3_ using an Agilent 1260 HPLC chromatograph (Agilent Technologies, USA) and Mixed-D (7.5 × 300 mm, 5 μm) or successively coupled Mixed-D and Mixed-E (7.5 × 300 mm, 3 μm) analytical columns with precolumns (both columns from Agilent Laboratories, USA). The nominal flow rate of the eluent was 1.0 and 0.7 mL min^–1^, respectively. The copolymer fractions were size separated on a Mixed-D analytical column with a precolumn. Sample concentrations and injected masses of samples on the column(s) were typically 1 mg mL^–1^ and 100 μg, respectively. Astra 7.3.1 software (Wyatt Technology Corp., USA) was used for data acquisition and evaluation.

### Liquid Adsorption Chromatography under Critical Conditions (LCCC)

A PLRP-S column with polystyrene-divinylbenzene (PS-DVB) as the reversed stationary phase (4.6 mm × 150 mm, 100 Å, 5 μm; Agilent Technologies, USA) was used for the isocratic LC experiments under critical conditions for P(POPA). The critical conditions for the P(POPA) homopolymers were at 25 °C at the mobile phase composition of THF: ACN = 15.6:84.4 vol %. The samples were dissolved in this critical composition mixture of ACN and THF and stirred overnight. The injected masses of the P(POPA) homopolymers and copolymers were 10 μg, while the injected mass of the PPO-1 homopolymer was 20 μg. Two detectors connected in series were used for detection, namely a UV detector (VWD) operating at 283 nm and an evaporative light scattering (ELS) detector 1260 Infinity (both Agilent Technologies, USA).

### LCCC Coupled with a Multidetection System (UV-MALS-RI)

Isocratic LCCC/UV-MALS-RI experiments under critical conditions for the P(POPA) were performed under the same conditions as LCCC, only the detection system was different. It consisted of UV, MALS, and RI detectors connected in series as in the SEC/UV-MALS-RI measurements. In LCCC/UV-MALS, the d*n*/d*c* needed to calculate the molar mass of the copolymers from the light scattering equation were calculated from the known d*n*/d*c* of the homopolymers and the known chemical composition of the copolymer fractions (determined from their ^1^H NMR spectra), according to (d*n*/d*c*)_copolymer_ = (d*n*/d*c*)_P(POPA)_·*wt*_P(POPA)_ + (d*n*/d*c*)_PPO_·*wt*_PPO_. The d*n*/d*c* values of the P(POPA) and PPO were determined off-line by measuring the RI of dialyzed homopolymer solutions of different concentrations (between 0.5 and 2.5 mg/mL, Table S2). The solutions of homopolymers were injected into the Optilab T-rEX RI detector using a Razel syringe pump model R99-E at a flow rate of 0.2 mL/min. The concentrations of the P(POPA) homopolymers and copolymers were followed with the UV detector at 283 nm and that of the PPO-1 homopolymer with the RI detector. The corresponding ε values of the homopolymers were determined assuming 100% mass recovery from the column, while the values of the copolymers were calculated from the ε values of the homopolymers and taking into account the copolymer chemical composition analogous to the d*n*/d*c* calculations. The injected masses of the P(POPA) homopolymers were 25 μg and those of the copolymers and PPO-1 were 100 μg. Astra 7.3.1 software (Wyatt Technology Corp., USA) was used for data acquisition and evaluation.

### Two-Dimensional Liquid Chromatography (LCCC × SEC 2D-LC)

The column used in the first LCCC dimension was PS-DVB (4.6 mm × 150 mm, 100 Å, 5 μm; Agilent Technologies, USA). The composition of the mobile phase (v/v %) in the first dimension was THF/ACN = 15.6:84.4 vol % and the flow rate was set to 0.04 mL min^–1^. The experiments were run at 25 °C (set by the thermostated oven), and the injected masses of the copolymers in the first dimension were typically 20 μg. In the second dimension, a SDV-linear M high-speed SEC column with linear porosity (20 mm × 50 mm I.D., particle size 5 μm, Polymer Standards Service, PSS GmbH, Germany) and THF as a mobile phase with a flow rate of 3 mL min^–1^ were used. The high-speed SDV column has a broad pore size distribution covering molar masses from 10^2^ to 10^6^ g mol^–1^. The SEC column was calibrated with eight PS standards with narrow molar mass distribution dissolved in THF at a concentration of typically 0.5 mg mL^–1^ and injected directly into the second dimension at a flow rate of 3.0 mL min^–1^.

### Fractionation of the Copolymers by LCCC on the PS-DVB Reverse Stationary Phase

The solutions of the copolymers in a mixture of THF/ACN = 15.6:84.4 vol % with a typical concentration of 1.5 mg mL^–1^ were separated using the PS-DVB reverse stationary phase. The high-performance LC fractionation system was equipped with a UV detector (VWD; operating at 283 nm), an RI detector, and an on-line fraction collector (all 1260 Agilent Technologies, USA). Experiments were performed under the conditions as described in the LCCC section. Solvents were removed from the collected fractions by rotary evaporation and further dried in a vacuum oven at 50 °C. Such isolated fractions were analyzed by ^1^H NMR and SEC/UV-MALS-RI to determine their chemical composition and molar mass characteristics.

### Gradient Liquid Adsorption Chromatography

For the gLAC experiments, a reversed-phase Zorbax Eclipse Plus C18 column (4.6 mm × 150 mm, 95 Å, 5 μm; Agilent Technologies, USA) or the same PLRP-S column as used for LCCC was used. Samples were dissolved in pure ACN and sample solutions were stirred overnight. The solvent gradient ran from 0 to 65% THF in ACN in 25 min on the C18 column and from 0 to 35% THF in ACN in 25 min on the PLRP-S column, and the column temperature was kept at 25 °C in both cases, maintained using a thermostated oven. The flow rate in the 1D gLAC was 1 mL min^–1^, and the masses of the samples injected onto the column ranged from 10 to 25 μg. The same detectors were used for the detection of the eluting species as in the LCCC.

## Results and Discussion

The copolymers of PO and POPA repeating units were prepared by simultaneous alternating one-pot ring-opening copolymerization of PO and phthalic anhydride (formation of A repeating units) and homopolymerization of PO (formation of B repeating units). The two-component organocatalyst system used provides good control over the molar mass characteristics and unique control over the chemical composition and microstructure of the copolymers depending on the ratio of Lewis basic and acidic catalytic components (Table S3, Figures S1–S5).^61−63^

**Figure 1:**
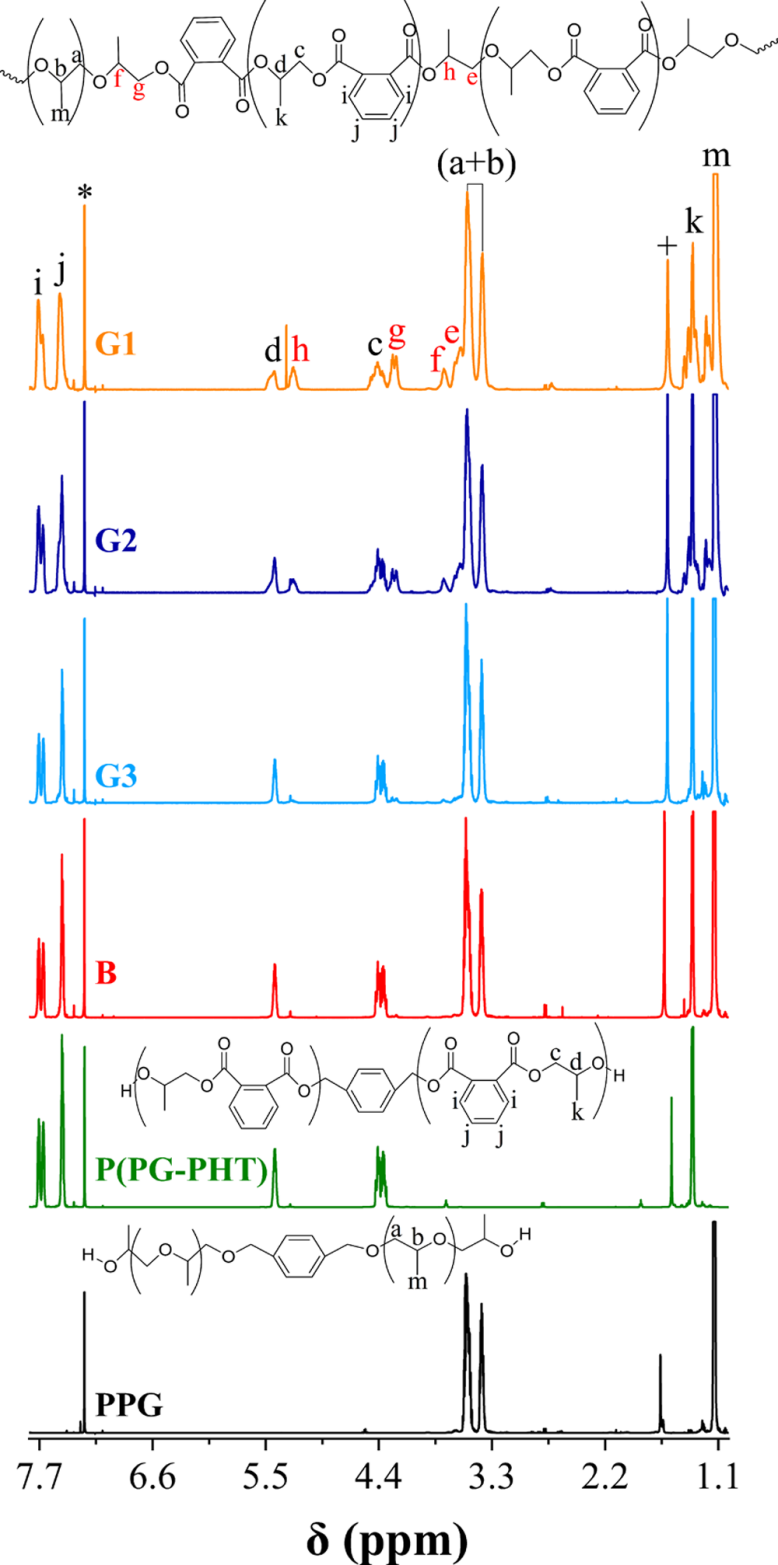
^1^H NMR spectra of PPO and P(POPA) homopolymers, PPO-*co*-P(POPA) copolymers with different gradient profiles. The CDCl_3_ residual peak and water in chloroform are marked with * and +, respectively.

In ^1^H NMR spectra, PPO homopolymers show overlapping proton signals of the CH_2_ and CH groups (b and a) in the range of δ 3.25–3.75 ppm, while P(POPA) homopolymers show proton signals of the CH_2_ and CH groups (c and d) adjacent to the ester group in the ranges of δ 4.30–4.45 ppm and δ 5.35–5.45 ppm, respectively (Figure 1). In addition, the P(POPA) homopolymers show aromatic proton signals (i and j) between δ 7.5 and 7.7 ppm. The P(PO-*co*-POPA) copolymers show proton signals of both P(POPA) and PPO segments, but in contrast to the homopolymers, they show additional signals e, h and g, f, which are due to the CH_2_ and CH groups of the ester-to-ether and ether-to-ester PO linking units, respectively. The intensity of these signals decreases from G1 to G3, which is consistent with the increase in gradient strength and thus a different microstructure that strongly influences the microphase separation and thermal behavior of the copolymers (Figure S6).

By comparing the integral ratio of the aromatic protons (i) of POPA with the methylene and methyne protons (a, b, e, and f) of the PO repeating units in the ^1^H NMR spectra, the average chemical composition of the copolymers was determined according to eq 1 (Table 1).
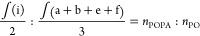
1

**Table 1:** Molar Mass Characteristics of the Copolymers and Their Individual Constituents Determined by SEC/UV-MALS-RI and Chemical Composition of the Copolymers (POPA: PO) Determined by SEC/UV-MALS-RI (SEC-3d) and ^1^H NMR

						POPA/PO [mol %]	
sample	*M̅*_w,PPO_ [kg/mol]	*M̅*_n,PPO_ [kg/mol]	*M̅*_w,P(POPA)_ [kg/mol]	*M̅*_n,P(POPA)_ [kg/mol]	*D̵*^a^ copolymer	SEC-3d	^1^H NMR
G1	14.5	13.8	18.2	16.4	1.32	25.1:74.9	24.1:75.9
G2	17.8	17.0	25.2	24.6	1.13	28.9:71.1	26.4:73.6
G3	18.3	17.1	22.6	20.6	1.07	25.3:74.7	23.9 76.1
B	15.4	15.2	25.0	22.8	1.03	29.7:70.3	27.8:72.2

a *D̵* = *M̅*_w_/*M̅*_n_ as obtained by experimentally determined d*n*/d*c* of the copolymer.

The SEC/-UV-MALS-RI allows not only the determination of the molar mass averages of the copolymers but also of their individual constituents and thus of the chemical composition^64,65^ because only the POPA repeating units are UV active at 283 nm, whereas the PO repeating units are not visible at this wavelength (Table 1, Figure S7). A prerequisite for such a determination is a narrow chemical composition distribution of copolymers because only then the apparent weight-average molar masses (*M*_w,app_) obtained by light scattering are in good agreement with the correct values.^66−69^ Because *M*_w,app_ of the copolymer heterogeneous in chain composition depends on the RI of the solvent used and this dependence is related to the degree of heterogeneity,^66,67^ the molar mass averages were measured in CHCl_3_ and THF. The *M*_w,app_ of the copolymers and their constituents obtained in the two solvents agree well (Table S4), indicating low heterogeneities in the composition of the PPO-*co*-P(POPA), as expected for controlled ring-opening polymerization. In addition, the copolymers exhibit a narrow distribution in molar mass, which is reflected in their low dispersity values (Table 1). The molar mass averages of the copolymers and their PPO and P(POPA) constituents are comparable between the samples, except for G1, which has slightly lower molar mass averages for both constituents. The chemical compositions of the copolymers determined by SEC/UV-MALS-RI from the number-average molar masses of both constituents are consistent with the chemical compositions of the copolymers determined by ^1^H NMR (Table 1).

### LCCC on the PS-DVB Reverse Stationary Phase

Because the P(POPA) constituent in G1 has a lower molar mass than in the other copolymers, while the molar mass of the PPO constituent is more comparable between the copolymers (Table 1), we first performed the separation of the copolymers at critical conditions for P(POPA), where the entropic and enthalpic contributions of the P(POPA) constituent compensate each other,^70^ so that the separation is solely governed by the molar mass of the PPO constituent and the microstructure of the copolymers. The critical conditions at which the P(POPA) homopolymers with different molar masses co-eluted on the PS-DVB reverse stationary phase at a temperature of 25 °C were determined to be at the THF/ACN mobile phase composition of 15.6:84.4 vol % (Figure S8, Table S1). The PPO-1 homopolymer with a number-average molar mass of 17.3 kg mol^–1^ eluted at the largest elution volume, while the PPO-2 with a number-average molar mass of 33.5 kg mol^–1^ retained in the column at the critical mobile phase composition, indicating a pronounced influence of molar mass on the elution of the PPO homopolymers.

In LCCC, all the copolymers elute from the PS-DVB column before the PPO-1 homopolymer because the copolymers also consist of the POPA repeat units, which reduces the probability of interaction of the PPO copolymer segments with the stationary phase. Because the PO sequences are distributed differently along the copolymer chains, it is expected that the probability of interaction of the PPO segments with the stationary phase, and thus the elution volume of the copolymers, depends not only on the molar mass of the PPO constituent but also on the average PPO segment length, which is defined by the microstructure. As expected, the elution of the gradient copolymers from the PS-DVB column occurs in the order of increasing gradient strength (Figure S8). The gradient copolymer with the strongest gradient (G3) elutes slightly after the block copolymer (B), although the intensity of the ^1^H NMR signals of the linking units is slightly higher in the former (Figure 1). This peculiar behavior is attributed to the somewhat higher total molar mass of the PPO constituent in G3 compared to B (17.1 vs 15.2 kg mol^–1^), which has a dominant effect on the elution over the copolymer microstructure. The width of the chromatographic peak is broadest for the PPO-1 homopolymer, which interacts most strongly with the stationary phase and is mainly associated with kinetically controlled diffusion processes.^71,72^ The copolymers show narrower peaks than the PPO-1 homopolymer. Their width decreases in the order G3 ∼ B, followed by G1 and finally G2, which is not consistent with decreasing gradient strength. Moreover, G1 and also G2 show asymmetric peaks with shoulders on the left peak sides, while G3 and B show additional low-intensity peaks baseline separated from the main copolymer peaks. Because the observed peculiarities could be a consequence of the distribution in the total molar mass of the PPO constituent and/or a distribution in the segment length (defined by the microstructure) of the PPO as a chromatographically visible copolymer constituent, the copolymers were further analyzed by LCCC/UV-MALS-RI and LCCC × SEC 2D-LC.

**Figure 2:**
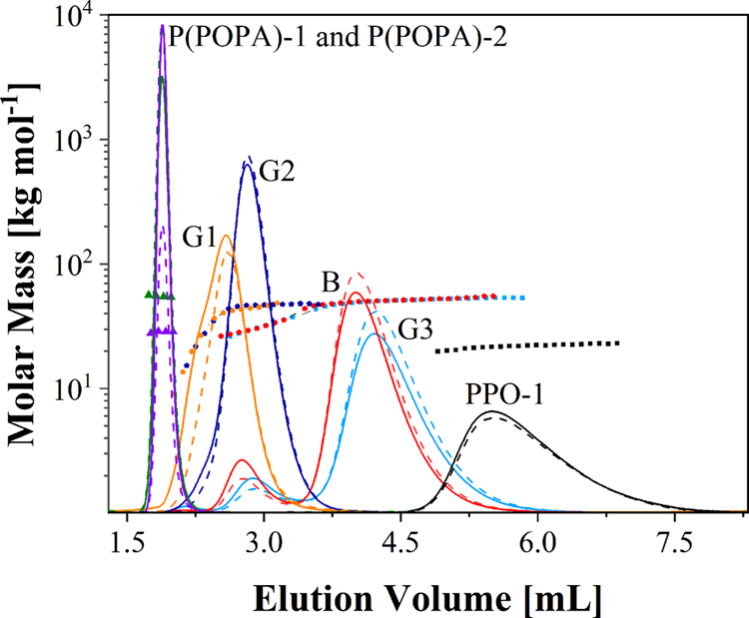
LCCC/UV-MALS-RI chromatograms on the PS-DVB reversed stationary phase for P(POPA)-1, P(POPA)-2, PPO-1 homopolymers, and their block and gradient copolymers with different gradient strengths. The composition of the mobile phase was THF/ACN = 15.6:84.4 vol % at 25 °C. The solid lines represent the RI detector response for PPO-1 and the UV detector responses for the other samples, the dashed lines represent the 90° light scattering (LS) detector responses, and the dotted lines show the molar mass as a function of elution volume.

Because LCCC is an isocratic method, it was coupled with the UV-MALS-RI detection system to determine molar mass as a function of elution volume by using UV as the concentration detector for the copolymers and the RI as the concentration detector for the PPO-1 homopolymer (Figure 2). Unfortunately, the molar masses of the individual constituents of the copolymers could not be determined over the whole copolymer peaks because the solvent peak partially overlapped with the chromatographic peaks of the copolymers in the RI chromatograms. Nevertheless, for each copolymer, the curves representing the copolymer molar mass as a function of elution volume determined by the UV-MALS-RI system, where d*n*/d*c* is calculated at each elution slice, and the average d*n*/d*c* of the copolymer agree well, which again confirms a narrow distribution in the chemical composition of the PPO-*co*-P(POPA) (Figure S8). The LCCC/UV-MALS experiments show that the early eluting low-intensity peaks of G3 and B and the shoulders on the left side of the chromatographic peaks of G1 and G2 are due to the presence of a small amount of low molar mass species in the copolymers, consisting of a lower total molar mass of the chromatographically visible PPO constituent compared to those in the main fractions of the copolymers. In addition, LCCC/UV-MALS provided us with important information that self-association of the copolymers, leading to high molar mass aggregates, is not the reason for the late elution of B and G3 compared to G1 and G2. This is evident from the molar masses of the copolymers determined by LCCC/UV-MALS-RI, which correlate well with those determined by SEC/UV-MALS-RI (Figure 2, Table 1).

**Figure 3:**
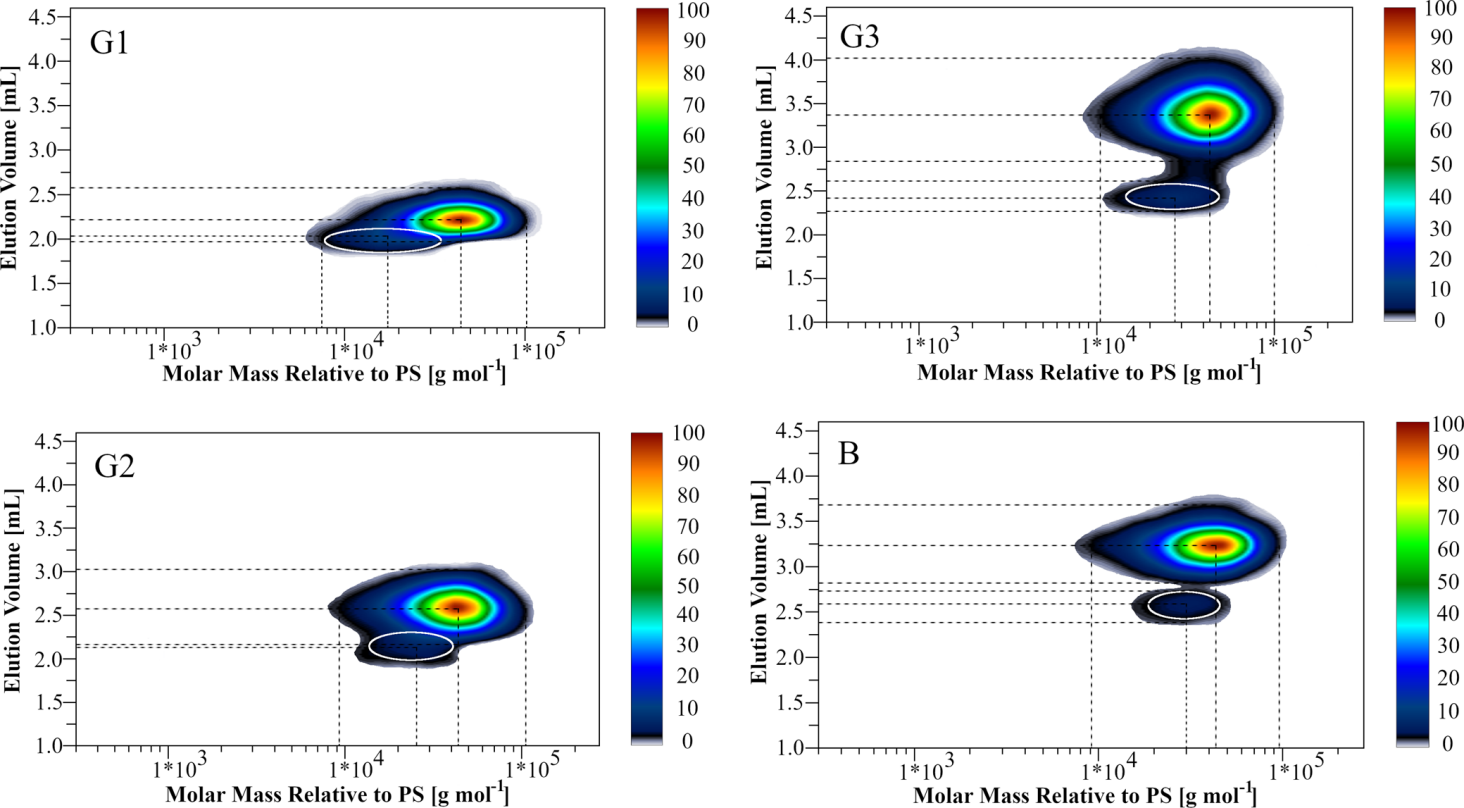
LCCC × SEC 2D-LC contour plots of the copolymers. First dimension (*y*-axis) LCCC: PS-DVB stationary phase; mobile phase: THF/ACN = 15.6:84.4 vol %; flow rate: 0.04 mL min^–1^; *T* = 25 °C. Second dimension (*x*-axis) SEC: SDV-M high-speed column calibrated with PS standards; mobile phase: THF; flow rate: 3 mL min^–1^; ELS detector. The color scale indicates the relative intensity of the ELS detector response.

The presence of low molar mass species in the samples was also confirmed by LCCC × SEC 2D-LC, where LCCC was combined with SEC to form a 2D online chromatography system to correlate the length of the PPO copolymer component and the microstructure of the species eluted from the first LCCC dimension (*y*-axis) with the relative molar mass determined in the second SEC dimension (*x*-axis). The contour plots of G1 and G2 show an asymmetric spot along both axes, while G3 and B show two spots with different intensities (Figure 3). The determination of the relative molar mass averages of each spot in the 2D-LC contour plots, by calibrating the second SEC dimension with PS standards, shows a lower molar mass of the early eluting species marked with white circles in Figure 3 (in LCCC observed as low-intensity peaks for B and G3 and as asymmetric peaks for G1 and G2) compared to the later eluting species (Table S5), which confirms the LCCC-MALS results.

The presence of low molar mass species is most likely due to transesterification between the terminal hydroxyl groups and the ester groups in the copolymer, leading to a broadening of the molar mass distribution by the formation of lower molar mass species. The highest content of low molar mass species in G1 is due to the difficulty in removing the traces of catalyst residues from the G1 viscous liquid sample and/or the fact that transesterification is favored for the copolymer with weaker gradient strength because the more widely distributed ester groups are more accessible. The presence of low molar mass species in the copolymers is also evident in the SEC/UV-MALS-RI chromatograms (Figure S7) as a slight tailing of the peaks on the low molar mass side, especially when SEC was run on two columns connected in series with improved resolution (Figure S9). Their content in the copolymers increases with decreasing gradient strength, which is reflected in increasing dispersity values of the copolymers in the same order (Table 1).

Because the copolymers contain small amounts of low molar mass species, they were fractionated into four fractions according to the scheme shown in Figure S10 to accurately determine the chemical composition and molar mass of the two copolymer constituents in each copolymer fraction of the LCCC chromatographic peaks by ^1^H NMR and SEC/UV-MALS-RI, respectively, to unambiguously evaluate the contributions of the molar mass of the PPO constituent and the average PPO segment length (microstructure) to the elution of the copolymers. The average length of the PPO segments in the copolymer fractions was determined from ^1^H NMR spectra of copolymers by comparing the intensities of the signals of PPO repeating units (denoted with a, b, e, and f) with the intensity of the signal due to the PO linking units (g) according to eq 2 (Table 2).
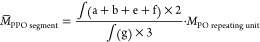
2

**Table 2:** Chemical Composition (POPA:PO) of the Copolymer Fractions Determined by ^1^H NMR and Molar Mass Characteristics of the Copolymer Fractions and Their P(POPA) and PPO Constituents Determined by SEC/UV-MALS-RI^a^

			Copolymer		P(POPA)		PPO			
	Fraction number	POPA/PO ^1^H NMR [mol %]	*M̅*_n_	*M̅*_w_ [kg/mol]	*M̅*_n_	*M̅*_w_ [kg/mol]	*M̅*_n_	*M̅*_w_ [kg/mol]	*M̅*_PPO,segment_ [g/mol]	*N̅*_PPO_
G1	1	24.5:75.5	12.6	18.1	6.3	10.1	6.2	8.0	353	17
	2	25.9:74.1	33.9	34.7	18.1	18.4	15.7	16.3	369	43
	3	26.0:74.0	35.4	35.6	18.9	19.1	**16.4**	**16.5**	**367**	**45**
	4	25.1:74.9	34.8	35.2	18.4	18.6	16.3	16.7	384	42
G2	1	27.4:72.6	28.7	32.0	15.7	19.8	11.8	12.2	446	26
	2	29.5:70.5	43.5	43.6	26.7	26.8	16.7	16.8	448	37
	3	28.7:71.3	41.7	41.8	24.7	24.8	**16.9**	**16.9**	**465**	**36**
	4	26.5:73.5	40.7	40.8	23.1	23.1	17.2	17.7	504	34
G3	1	23.9:76.1	33.9	34.1	21.8	22.0	12.0	12.1	1757	7
	2	27.2:72.8	40.8	40.9	23.8	24.0	16.4	16.9	1632	10
	3	27.1:72.9	41.4	41.4	24.7	24.7	**16.7**	**16.7**	**1736**	**10**
	4	25.4:74.6	40.4	42.2	21.3	22.5	16.6	19.7	1888	9
B	1	25.0:75.0	32.5	32.9	21.0	21.3	11.4	11.6	5700^b^	2
	2	29.2:70.8	39.5	39.9	22.0	24.4	15.2	15.5	7600^b^	2
	3	29.7:70.3	42.5	42.8	26.1	26.3	**15.7**	**16.5**	**7850**^b^	**2**
	4	28.7:71.3	41.7	42.3	25.3	25.7	15.4	16.6	7700^b^	2

a The copolymer fractions were collected at the outlet of the PS-DVB column under critical conditions for P(POPA) according to the scheme shown in Figure S10.

b Because of the low intensity of the ^1^H NMR signals of the ester-to-ether PO linking units, *M̅*_PPO,segment_ was calculated from the *M̅*_n,PPO_ determined by SEC/UV-MALS-RI and taking into account that B is a triblock copolymer with two PPO blocks on each side of the copolymer chain.

The average number of PPO segments (*N̅*_PPO_) in each fraction of the gradient copolymers was determined according to eq 3 by using the results of the number-average molar mass of the PPO constituent in the fractions of the gradient copolymers (*M̅*_n, PPO_) determined by SEC/UV-MALS-RI and the average length of the PPO segments (*M̅*_PPO, segment_) determined by ^1^H NMR (Table 2).
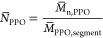
3

The fractionation results confirmed our previous assumption that the microstructure affects the separation of the gradient copolymers, as the total molar mass of the PPO constituent in the main fractions of the gradient copolymers is comparable, but their elution volume is different, suggesting that the average PPO segment length contributes significantly to the elution behavior of the gradient copolymers. Interestingly, the G3 with the strongest gradient elutes slightly after the block copolymer from the PS-DVB column, although its average PPO segment length is shorter than that of the block copolymer, suggesting that in this case, the total average molar mass of the PPO constituent in the copolymer dominates the elution behavior over the microstructure.

In addition, the fractionation again confirmed that the early eluting fractions of the copolymers are of lower molar masses compared to the later eluting fractions, including the molar mass of the PPO constituent (Table 2). The lower resolution between the low and high molar mass species in G1 and G2 compared to B and G3, where the low molar mass species are baseline separated from the main chromatographic peaks, is also a consequence of the different microstructure of these copolymers. Namely, B and G3 show a more ordered sequence distribution of comonomer units along the copolymer chains than G1 and G2. Further evidence that the microstructure has an important influence on the elution behavior of the copolymers is the fact that the low molar mass fractions of B and G3 practically co-eluted with the main high molar mass fraction of G2, although the molar mass of the total PPO copolymer constituent in G2 is higher by ∼5–6 kg mol^–1^.

### gLAC on the PS-DVB and C18 Reverse Stationary Phases

**Figure 4:**
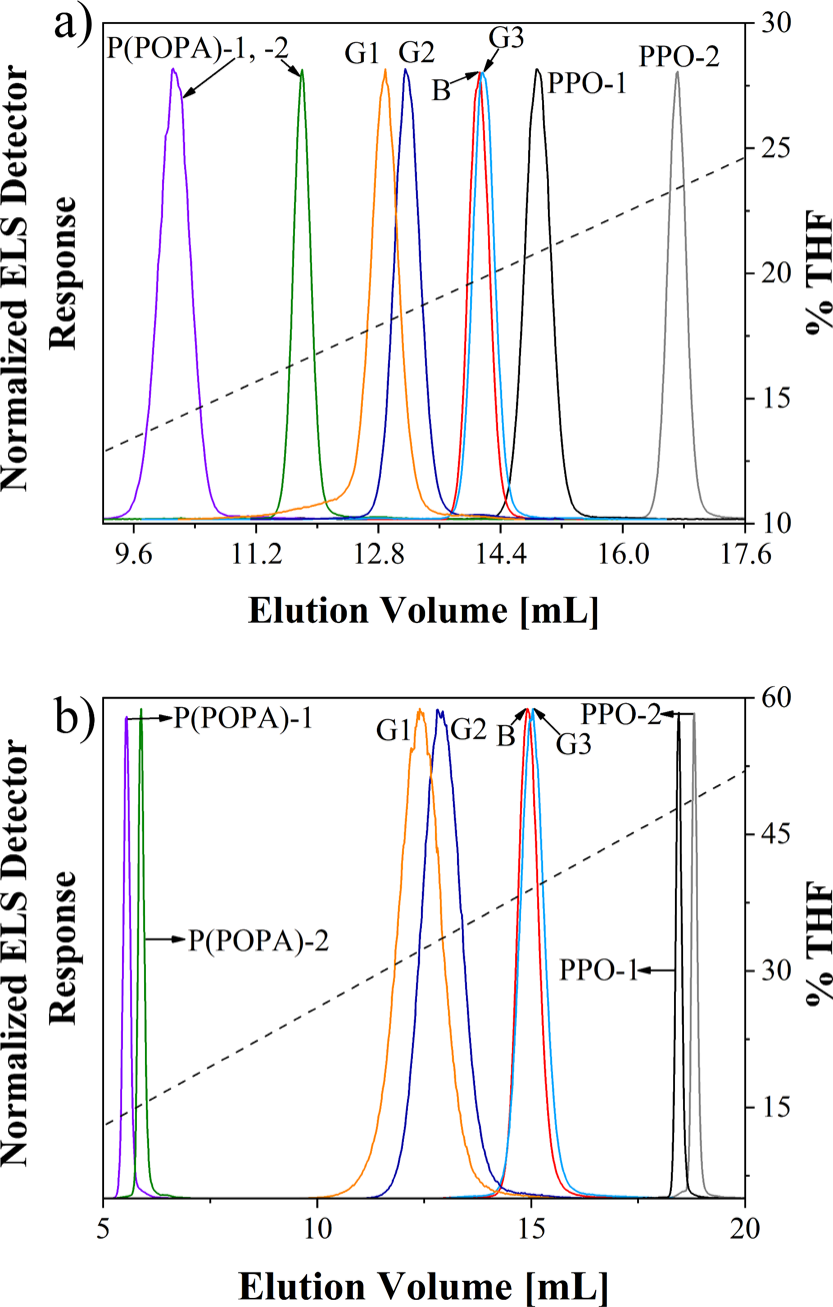
gLAC chromatograms of P(POPA) and PPO homopolymers, block copolymer, and gradient copolymers with different gradient strengths obtained on (a) PS-DVB column (solvent gradient: 0–35% THF in ACN in 25 min) and (b) C18 column (solvent gradient: 0–65% THF in ACN in 25 min).

The elution behavior of PPO and P(POPA) homopolymers, both with two different molar masses and narrow molar mass distributions, and PPO-*co*-P(POPA) copolymers, was also studied under gLAC conditions on the PS-DVB and the C18 reverse stationary phase. On both columns, the PPO homopolymers retain longer than the P(POPA) homopolymers, which is attributed to the stronger interaction of PPO with the stationary phases (Figure 4). However, a much stronger effect of molar mass on the retention time for a given homologous series was observed on the PS-DVB than on the C18 reverse stationary phase, where it was almost negligible. These results show that a critical molar mass, above which it no longer has a significant effect on elution time, is reached at lower molar masses on the C18 than on the PS-DVB column, with which the homopolymers interact more weakly and therefore require a weaker mobile phase (25 vs 53 vol % THF in ACN) to desorb them from the column.

PPO-*co*-P(POPA) copolymers elute from both columns between the two types of homopolymers in the same order as observed in LCCC, only the resolution between G1 and G2 and especially between G3 and B is inferior. Moreover, low molar mass species co-eluted with high molar mass species, so that no additional peaks are observed for G3 and B, while the peak of G2 is more symmetrical. These results are explained by the fact that the molar mass of the more strongly interacting PPO copolymer constituent in gLAC has a smaller effect on copolymer elution than in LCCC and that the chemical composition of the low molar mass species in G3 and B is in favor of the PPO units (Table 2). The only exception is G1 analyzed on the PS-DVB column, which shows a pronounced peak tailing on the left peak side and which contains a fraction with the lowest molar mass in the largest amount among all copolymers. The width of the copolymer peaks in the gLAC chromatograms does not match that observed in LCCC. In fact, the peak width in gLAC increases with decreasing gradient strength and thus correlates with dispersity in molar mass (Figure 4 and Table 1). However, the influence of dispersity in chemical composition and microstructure cannot be neglected, as shown below.

The G1 and G2 copolymers were fractionated on a C18 column according to the scheme shown in Figure S11, and the collected fractions were analyzed by SEC/UV-MALS-RI and ^1^H NMR (Table S6). The fractionation results of G1 and G2 show that the chemical composition changes a bit with increasing elution volume in favor of the PO sequences, indicating slight chemical heterogeneity of G1 and G2 copolymers. The chemical composition of the third and fourth fractions of G1 is in favor of the more interacting PPO segments compared to the corresponding fractions in G2. Moreover, the molar mass of these fractions is higher in G2 than in G1, but mainly at the expense of the P(POPA) constituent, which interacts to a lesser degree with the stationary phase, while the molar mass of the more strongly interacting PPO constituent is more comparable. From these results, we conclude that the microstructure has a decisive influence on the earlier elution of G1 compared to G2. On the other hand, the later elution of G3 compared to B is much less noticeable in gLAC than in LCCC and is attributed to the chemical composition favoring the PPO constituent and the higher molar mass of the more strongly interacting PPO constituent in G3 than in B (Table 1). All these parameters dominate the elution over the microstructure in these two copolymers.

## Conclusions

The focus of our work was to investigate the influence of microstructure on the chromatographic behavior of copolymers. To this end, we studied PPO and P(POPA) homopolymers with different molar masses and narrow molar mass distribution, and their block and gradient copolymers with different gradient strengths but comparable molar masses and chemical compositions using C18 and PS-DVB reversed stationary phases. On both stationary phases, the gradient copolymers eluted from the column according to the gradient strength, demonstrating the crucial role of microstructure in their elution behavior. However, the contributions of molar mass and especially chemical composition in gLAC and total molar mass of the chromatographically visible copolymer constituent in LCCC should not be overlooked, as shown in our case by the elution behavior of the block copolymer and the gradient copolymer with the strongest gradient. Because the microstructure, among other parameters, affects the net chain interactivity of the gradient copolymers, the results obtained with interactive liquid chromatographic techniques for the copolymers should be interpreted carefully.

## Supporting Information Available

Synthesis of PPO-*co*-P(POPA) copolymers and corresponding homopolymers, calculation procedure in SEC/UV-MALS-RI, fractionation of the copolymers on the C18 column, DSC, results of synthesis, DSC, SEC/UV-MALS-RI, LCCC, and 2D-LC, schematic representation of fraction collection, and fractionation results on the C18 column (PDF)

## References

[ref1] Philipsen H. J. A. Determination of chemical composition distributions in synthetic polymers. J. Chromatogr. A 2004, 1037, 329–350. 10.1016/j.chroma.2003.12.047. 15214674

[ref2] Pasch H. Advanced fractionation methods for the microstructure analysis of complex polymers. Polym. Adv. Technol. 2015, 26, 771–784. 10.1002/pat.3479.

[ref3] Cools P. J. C. H. Characterization of Copolymers by Gradient Elution Chromatography; Mobach B., Ed.; Technische Universiteit Eindhoven: Eindhoven, 1999; pp 1–3.

[ref4] Mori S.; Uno Y.; Suzuki M. Separation of Styrene–Methyl Methacrylate Random Copolymers According to Chemical Composition and Molecular Size by Liquid Adsorption and Size Exclusion Chromatography. Anal. Chem. 1986, 58, 303–307. 10.1021/ac00293a009.

[ref5] Sato H.; Takeuchi H.; Tanaka Y. Analysis of Chemical Composition Distribution of Styrene-Methyl Methacrylate by High-Performance Liquid Chromatography. Macromolecules 1986, 19, 2613–2617. 10.1021/ma00164a026.

[ref6] Schunk T. C. Composition distribution separation of methyl methacrylate-matacrylic acid copolymers by normal-phase gradient elution high-performance liquid chromatography. J. Chromatogr. A 1994, 661, 215–226. 10.1016/0021-9673(94)85192-1.

[ref7] Brun Y.; Alden P. Gradient separation of polymers at critical point of adsorption. J. Chromatogr. A 2002, 966, 25–40. 10.1016/S0021-9673(02)00705-7. 12214702

[ref8] Schultz R.; Engelhardt H. HPLC of Synthetic Polymers II. Chromatographic Characterization of Copolymers from Styrene and Acrylonitrile. Chromatographia 1990, 29, 325–332. 10.1007/BF02261298.

[ref9] Macko T.; Arndt J.-H.; Brüll R. HPLC Separation of Ethylene-Vinyl Acetate Copolymers According to Chemical composition. Chromatographia 2019, 82, 725–732. 10.1007/s10337-019-03697-x.

[ref10] Lyons J. W.; Poche D.; Wang F. C.-Y.; Smith P. B. Recent Advances in Polymer Separations. Adv. Mater. 2000, 12, 1847–1854. 10.1002/1521-4095(200012)12:23<1847::AID-ADMA1847>3.0.CO;2-F.

[ref11] Falkenhagen J.; Much H.; Stauf W.; Müller A. H. E. Characterization of Block Copolymers by Liquid Adsorption Chromatography at Critical Conditions. 1. Diblock Copolymers. Macromolecules 2000, 33, 3687–3693. 10.1021/ma991903j.

[ref12] Malik M. I.; Sinha P.; Bayley G. M.; Mallon P. E.; Pasch H. Characterization of Polydimethylsiloxane-block-polystyrene (PDMS-b-PS) Copolymers by Liquid Chromatography at Critical Conditions. Macromol. Chem. Phys. 2011, 212, 1221–1228. 10.1002/macp.201000711.

[ref13] Tufail M. K.; Abdul-Karim R.; Rahim S.; Musharraf S. G.; Malik M. I. Analysis of individual block length of amphiphilic di-& tri-block copolymers containing poly(ethylene oxide) and poly(methyl methacrylate). RSC Adv. 2017, 7, 41693–41704. 10.1039/C7RA08804C.

[ref14] Malik M. I.; Mahboob T.; Ahmed S. Characterization of poly(2-vinylpyridine)-block-poly(methyl methacrylate) copolymers and blends of their homopolymers by liquid chromatogrpahy at critical conditions. Anal. Bioanal. Chem. 2014, 406, 6311–6317. 10.1007/s00216-014-8075-2. 25116604

[ref15] Hehn M.; Maiko K.; Pasch H.; Hiller W. Online HPLC-NMR: An Efficient Method for the Analysis of PMMA with Respect to Tacticity. Macromolecules 2013, 46, 7678–7686. 10.1021/ma401429n.

[ref16] Kitayama T.; Janco M.; Ute K.; Niimi R.; Hatada K.; Berek D. Analysis of Poly(ethyl methacrylate)s by On-Line Hyphenation of Liquid Chromatogrpahy at the Critical Adsorption Point and Nuclear Magnetic Resonance Spectroscopy. Anal. Chem. 2000, 72, 1518–1522. 10.1021/ac991065r. 10763248

[ref17] Hiller W.; Sinha P.; Pasch H. On-Line HPLC-NMR of PS-b-PMMA and Blends of PS and PMMA: LCCC-NMR at Critical Conditions of PS. Macromol. Chem. Phys. 2007, 208, 1965–1978. 10.1002/macp.200700166.

[ref18] Hiller W.; Pasch H.; Sinha P.; Wagner T.; Thiel J.; Wagner M.; Müllen K. Coupling of NMR and Liquid Chromatography at Critical Conditions: A New Tool for the Block Length and Microstructure Analysis of Block Copolymers. Macromolecules 2010, 43, 4853–4863. 10.1021/ma902359e.

[ref19] Hiller W.; Sinha P.; Hehn M.; Pasch H.; Hofe T. Separation Analysis of Polyisoprenes Regarding Microstructure by Online LCCC-NMR and SEC-NMR. Macromolecules 2011, 44, 1311–1318. 10.1021/ma102750s.

[ref20] Sinha P.; Hiller W.; Pasch H. Characterization of blends of polyisoprene and polystyrene by on-line hyphenation of HPLC and ^1^H-NMR: LC-CC-NMR at critical conditions for both homopolymers. J. Sep. Science 2010, 33, 3494–3500. 10.1002/jssc.201000486. 20931613

[ref21] Sinha P.; Hiller W.; Bellas V.; Pasch H. Analysis of polystyrene-b-polyisoprene copolymers by coupling of liquid chromatography at critical conditions to NMR at critical conditions of polystyrene and polyisoprene. J. Sep. Sci. 2012, 35, 1731–1740. 10.1002/jssc.201101033. 22707375

[ref22] Chang T. Chromatographic Separation of Polymers. Recent Progress in Separation of Macromolecules and Particulates; Wang Y.; Gao W.; Orski S.; Liu X. M., Eds.; American Chemical Society: Washington, 2018; pp 1–17.

[ref23] Lee H. C.; Chang T. Polymer molecular weight characterization by temperature gradient high performance liquid chromatography. Polymer 1996, 37, 5747–5749. 10.1016/S0032-3861(96)00510-1.

[ref24] Lee W.; Lee H.; Cha J.; Chang T.; Hanley K. J.; Lodge T. P. Molecular Weight Distribution of Polystyrene Made by Anionic Polymerization. Macromolecules 2000, 33, 5111–5115. 10.1021/ma000011c.

[ref25] Cong R.; deGroot W.; Parrott A.; Yau W.; Hazlitt L.; Brown R.; Miller M.; Zhou Z. A New Technique for Characterizing Comonomer Distribution in Polyolefins: High-Temperature Thermal Gradient Interaction Chromatography (HT-TGIC). Macromolecules 2011, 44, 3062–3072. 10.1021/ma200304e.

[ref26] Alam M. M.; Jack K. S.; Hill D. J. T.; Whittaker A. K.; Peng H. Gradient Copolymers – Preparation, properties and practice. Eur. Polym. J. 2019, 116, 394–414. 10.1016/j.eurpolymj.2019.04.028.

[ref27] Mok M. M.; Kim J.; Torkelson J. M. Gradient Copolymers with Broad Glass Transition Temperature Regions: Design of Purely Interphase Compositions for Damping Applications. J. Polym. Sci., Part B: Polym. Phys. 2008, 46, 48–58. 10.1002/polb.21341.

[ref28] Zhang J.; Farias-Mancilla B.; Destarac M.; Schubert U. S.; Keddie D. J.; Guerrero-Sanchez C.; Harrisson S. Asymmetric Copolymers: Synthesis, Properties, and Applications of Gradient and Other Partially Segregated Copolymers. Macromol. Rapid Commun. 2018, 39, 1800357 10.1002/marc.201800357. 30221423

[ref29] Beginn U. Gradient Copolymers. Colloid Polym. Sci. 2008, 286, 1465–1474. 10.1007/s00396-008-1922-y.

[ref30] Mok M. M.; Kim J.; Wong C. L. H.; Marrou S. R.; Woo D. J.; Dettmer C. M.; Nguyen S. T.; Ellison C. J.; Shull K. R.; Torkelson J. M. Glass Transition Breadths and Composition Profiles of Weakly, Moderately, and Strongly Segregating Gradient Copolymers: Experimental Results and Calculations from Self-Consistent Mean-Field Theory. Macromolecules 2009, 42, 7863–7876. 10.1021/ma9009802.

[ref31] Zaremski M. Y.; Kalugin D. I.; Golubev V. B. Gradient Copolymers: Synthesis, Structure and Properties. Polym. Sci., Ser. A 2009, 51, 103–122. 10.1134/S0965545X09010088.

[ref32] Zheng C.; Huang H.; He T. Micellization of St/MMA Gradient Copolymers: A General Picture of Structural Transitions in Gradient Copolymer Micelles. Macromol. Rapid Commun. 2013, 34, 1654–1661. 10.1002/marc.201300553. 24027091

[ref33] Chen J.; Li J.-J.; Luo Z.-H. Synthesis, Surface Property, Micellization and pH Responsivity of Fluorinated Gradient Copolymers. J. Polym. Sci., Part A: Polym. Chem. 2013, 51, 1107–1117. 10.1002/pola.26473.

[ref34] Chen Y.; Zhang Y.; Wang Y.; Sun C.; Zhang C. Synthesis, Characterization, and Self-Assembly of Amphiphilic Fluorinated Gradient Copolymer. J. Appl. Polym. Sci. 2013, 127, 1485–1492. 10.1002/app.37556.

[ref35] Kravchenko V. S.; Potemkin I. I. Micelles of Gradient vs Diblock Copolymers: Difference in the Internal Structure and Properties. J. Phys. Chem. B 2016, 120, 12211–12217. 10.1021/acs.jpcb.6b10120. 27933941

[ref36] Zhao Y.; Luo Y.-W.; Li B.-G.; Zhu S. pH Responsivity and Micelle Formation of Gradient Copolymers of Methacrylic Acid and Methyl Methacrylate in Aqueous Solution. Langmuir 2011, 27, 11306–11315. 10.1021/la2011875. 21819117

[ref37] Harrisson S.; Ercole F.; Muir B. W. Living spontaneous gradient copolymers of acrylic acid and styrene: one-pot synthesis of pH-responsive amphiphiles. Polym. Chem. 2010, 1, 326–332. 10.1039/B9PY00301K.

[ref38] Černochová Z.; Bogomolova A.; Borisova O. V.; Filippov S. K.; Černoch P.; Billon L.; Borisov O. V.; Štěpánek P. Thermodynamics of the multi-stage self-assembly of pH-sensitive gradient copolymers in aqueous solutions. Soft Matter 2016, 12, 6788–6798. 10.1039/C6SM01105E. 27451979

[ref39] Seno K.-I.; Tsujimoto I.; Kanaoka S.; Aoshima S. Synthesis of Various Stimuli-Responsive Gradient Copolymers by Living Cationic Polymerization and Their Thermally or Solvent Induced Association Behavior. J. Polym. Sci., Part A: Polym. Chem. 2008, 46, 6444–6454. 10.1002/pola.22953.

[ref40] Steinhauer W.; Hoogenboom R.; Keul H.; Moeller M. Block and Gradient Copolymers of 2-Hydroxyethyl Acrylate and 2-Methoxyethyl Acrylate via RAFT: Polymerization Kinetics, Thermoresponsive Properties, and Micellization. Macromolecules 2013, 46, 1447–1460. 10.1021/ma302606x.

[ref41] Seno K.-I.; Tsujimoto I.; Kikuchi T.; Kanaoka S.; Aoshima S. Thermosensitive Gradient Copolymers by Living Cationic Polymerization: Semibatch Precision Synthesis and Stepwise Dehydration-Induced Micellization and Physical Gelation. J. Polym. Sci., Part A: Polym. Chem. 2008, 46, 6151–6164. 10.1002/pola.22926.

[ref42] Zheng C.; Huang H.; He T. Gradient Structure-Induced Temperature Responsiveness in Styrene/Methyl Methacrylate Gradient Copolymers Micelles. Macromol. Rapid Commun. 2014, 35, 309–316. 10.1002/marc.201300769. 24353194

[ref43] Gil E.; Hudson S. Stimuli-responsive polymers and their bioconjugates. Prog. Polym. Sci. 2004, 29, 1173–1222. 10.1016/j.progpolymsci.2004.08.003.

[ref44] Okabe S.; Seno K.-i.; Kanaoka S.; Aoshima S.; Shibayama M. Micellization Study on Block and Gradient Copolymer Aqueous Solutions by DLS and SANS. Macromolecules 2006, 39, 1592–1597. 10.1021/ma052334k.

[ref45] Shull K. R. Interfacial Activity of Gradient Copolymers. Macromolecules 2002, 35, 8631–8639. 10.1021/ma020698w.

[ref46] Pandav G.; Pryamitsyn V.; Gallow K. C.; Loo Y.-L.; Genzer J.; Ganesan V. Phase behavior of gradient copolymer solutions: a Monte Carlo simulation study. Soft Matter 2012, 8, 6471–6482. 10.1039/C2SM25577D.

[ref47] Gallow K. C.; Jhon Y. K.; Tang W.; Genzer J.; Loo Y.-L. Cloud Point Suppression in Dilute Solutions of Model Gradient Copolymers with Prespecified Composition Profiles. J. Polym. Sci., Part B: Polym. Phys. 2011, 49, 629–637. 10.1002/polb.22226.

[ref48] Gallow K. C.; Jhon Y. K.; Genzer J.; Loo Y.-L. Influence of gradient strength and composition profile on the onset of the cloud point transition in hydroxyethyl methacrylate/dimethylaminoethyl methacrylate gradient copolymers. Polymer 2012, 53, 1131–1137. 10.1016/j.polymer.2012.01.027.

[ref49] Lefebvre M. D.; Olvera de la Cruz M.; Shull K. R. Phase Segregation in Gradient Copolymer Melts. Macromolecules 2004, 37, 1118–1123. 10.1021/ma035141a.

[ref50] Jiang R.; Jin Q.; Li B.; Ding D.; Wickham R. A.; Shi A.-C. Phase Behavior of Gradient Copolymers. Macromolecules 2008, 41, 5457–5465. 10.1021/ma8002517.

[ref51] Ganesan V.; Kumar N. A.; Pryamitsyn V. Blockiness and Sequence Polydispersity Effects in the Phase Behavior and Interfacial Properties of Gradient Copolymers. Macromolecules 2012, 45, 6281–6297. 10.1021/ma301136y.

[ref52] Alshehri I. H.; Pahovnik D.; Žagar E.; Shipp D. A. Stepwise Gradient Copolymers of n-Butyl Acrylate and Isobornyl Acrylate by Emulsion RAFT Copolymerizations. Macromolecules 2022, 55, 391–400. 10.1021/acs.macromol.1c01897.

[ref53] Brun Y. The mechanism of copolymer retention in interactive polymer chromatography. I. Critical point of adsorption for statistical copolymers. J. Liq. Chromatogr. Relat. Technol. 1999, 22, 3027–3065. 10.1081/JLC-100102075.

[ref54] Skvortsov A.; Trathnigg B. Martin’s rule revisited. Its molecular sense and limitations. J. Chromatogr. A 2003, 1015, 31–42. 10.1016/S0021-9673(03)01207-X. 14570317

[ref55] Zhu Y.; Ziebarth J.; Macko T.; Wang Y. How Well Can One Separate Copolymers According to Both Chemical Compositions and Sequence Distributions?. Macromolecules 2010, 43, 5888–5895. 10.1021/ma1007336.

[ref56] Trazkovich A. J.; Wendt M. F.; Hall L. M. Effect of Copolymer Sequence on Local Viscoelastic Properties near a Nanoparticle. Macromolecules 2019, 52, 513–527. 10.1021/acs.macromol.8b02136.

[ref57] Brun Y.; Foster P. Characterization of synthetic copolymers by interaction polymer chromatography: Separation by microstructure. J. Sep. Sci. 2010, 33, 3501–3510. 10.1002/jssc.201000572. 20949502

[ref58] Peltier R.; Bialek A.; Kuroki A.; Bray C.; Martin L.; Perrier S. Reverse-phase high performance liquid chromatography (RP-HPLC) as a powerful tool to characterize complex water-soluble copolymer architectures. Polym. Chem. 2018, 9, 5511–5520. 10.1039/C8PY00966J.

[ref59] Augenstein M.; Müller M. A. Gradient high performance liquid chromatography of polymers, 2^a)^ Characterization of block copolymers of decyl and methyl methacrylate synthesized via group transfer polymerization^b)^. Makromol. Chem. 1990, 191, 2151–2172. 10.1002/macp.1990.021910917.

[ref60] Cheruthazhekatt S.; Pasch H. Improved chemical composition separation of ethylene–propylene random copolymers by high-temperature solvent gradient interaction chromatography. Anal. Bioanal. Chem. 2013, 405, 8607–8614. 10.1007/s00216-013-7252-z. 23907688

[ref61] Li H.; He G.; Chen Y.; Zhao J.; Zhang G. One-Step Approach to Polyester-Polyether Block Copolymers Using Highly Tunable Biocomponent Catalyst. ACS Macro Lett. 2019, 8, 973–978. 10.1021/acsmacrolett.9b00439. 35619475

[ref62] Chen Y.; Shen J.; Liu S.; Zhao J.; Wang Y.; Zhang G. High Efficiency Organic Lewis Pair Catalyst for Ring-Opening Polymerization of Epoxides with Chemoselectivity. Macromolecules 2018, 51, 8286–8297. 10.1021/acs.macromol.8b01852.

[ref63] Li H.; Zhao J.; Zhang G. Self-Buffering Organocatalysis Tailoring Alternating Polyester. ACS Macro Lett. 2017, 6, 1094–1098. 10.1021/acsmacrolett.7b00654. 35650948

[ref64] Šmigovec Ljubič T.; Rebolj K.; Pahovnik D.; Hadjichristidis N.; Žigon M.; Žagar E. Utility of Chromatographic and Spectroscopic Techniques for a Detailed Characterization of Poly(styrene-b-isoprene) Miktoarm Star Copolymers with Complex Architecture. Macromolecules 2012, 45, 7574–7582. 10.1021/ma3012366.

[ref65] Haidar Ahmad I. A.; Striegel A. M. Determining the absolute, chemical-heterogeneity-corrected molar mass averages, distribution, and solution conformation of random copolymers. Anal. Bioanal. Chem. 2010, 396, 1589–1598. 10.1007/s00216-009-3320-9. 20012904

[ref66] Bushuk W.; Benoit H. Light-scattering studies of copolymers. I. Effect of heterogeneity of chain composition on the molecular weight. Can. J. Chem. 1958, 36, 1616–1626. 10.1139/v58-235.

[ref67] Kratochvíl P. Classical Light Scattering from Polymer Solutions; Elsevier: Amsterdam, 1987.

[ref68] Chau T. C.; Rudin A. Characterization of styrene-butadiene copolymers by light scattering. Polymer 1974, 15, 593–598. 10.1016/0032-3861(74)90159-1.

[ref69] Berry G. C. In Soft-Matter Characterization; Borsali R.; Pecora R., Eds.; Springer: New York, 2008; pp 41–131.

[ref70] Pasch H.; Trathnigg B. Multidimensional HPLC of Polymers; Alig I.; Pasch H., Eds.; Springer-Verlag: Berlin Heidelberg, 2013; pp 30.

[ref71] Berek D. Critical assessment of “critical” liquid chromatography of block copolymers. J. Sep. Sci. 2016, 39, 93–101. 10.1002/jssc.201500956. 26462613

[ref72] Striegel A. M. Method development in interaction polymer chromatography. Trends Anal. Chem. 2020, 130, 115990 10.1016/j.trac.2020.115990. PMC7919746 33654335

