# Supplementary material for: Influence of Microstructure on the Elution Behavior of Gradient Copolymers in Different Modes of Liquid Interaction Chromatography
Source: Anal Chem. 2022 May 23;94(22):7844–52. doi: 10.1021/acs.analchem.2c00193 (PMC9178556; doi:10.1021/acs.analchem.2c00193)
Supplement: Supplementary file 1 — ac2c00193_si_001.pdf [file ac2c00193_si_001.pdf]

Supporting Information

Influence of Microstructure on the Elution Behavior of Gradient Copolymers in Different Modes of Liquid Interaction Chromatography

Blaž Zdovc,[1,#] Heng Li,[2,#] Junpeng Zhao,[2,] David Pahovnik,[1] and Ema Žagar[1,*]*

[1]Department of Polymer Chemistry and Technology, National Institute of Chemistry, Hajdrihova 19, Ljubljana SI-1000, Slovenia

[2] Faculty of Materials Science and Engineering, South China University of Technology,

381 Wushan Road, Guangzhou 510641, P. R. China

*Corresponding authors:

Ema Žagar; Email: ema.zagar@ki.si, Phone: +386 1 4760 203

Junpeng Zhao; Email: msjpzhao@scut.edu.cn

Number of pages: 21

Number of figures: 11

Number of tables: 6

Table of Contents

List of Figures S2
List of Tables S4
Experimental Section S5
Chemicals for synthesis S5
Synthesis of PPO homopolymers..S5
Synthesis of alternating copolymers of PA and PO (referred to as P(POPA) homopolymers) S5
Synthesis of PPO-co-P(POPA) copolymers S6
Calculation Procedure in SEC/UV-MALS-RI..S6
Fractionation of the copolymers by gLAC on the C18 reverse-stationary phase S7
Differential scanning calorimetry (DSC)..S7
Results and Discussion S7

List of Figures

Figure S 1. Time dependence of the conversion of PA in the formation of POPA repeating units (or P(POPA) segments) (black triangles) and the conversion of PO in the formation of PPO segments (blue squares) during the copolymerization. S10

Figure S 2. ^{1}H NMR spectra of the aliquots withdrawn at different reaction times from the copolymerization of PA and PO performed in THF at RT with BDM as the initiator and the feed ratio of $[PA]_0/[PO]_0/[OH]_0/[tBuP_1]/[Et_3B]$ being 75/325/1/0.5/0.15, resulting in a block copolymer (B in Table 1). Conversions of PA and PO in Table 1 are calculated as a/(a+a') and d/(d+3e-3a'/4). S11

Figure S 3. ^{1}H NMR spectra of the aliquots withdrawn at different reaction times from the copolymerization of PA and PO performed in THF at RT with BDM as the initiator and the feed ratio of $[PA]_0/[PO]_0/[OH]_0/[tBuP_1]/[Et_3B]$ being 75/325/1/0.5/1.5, resulting in a gradient copolymer (G1 in Table 1). Conversions of PA and PO in Table 1 are calculated as a/(a+a') and d/(d+3e-3a'/4). S12

Figure S 4. ^{1}H NMR spectra of the aliquots withdrawn at different reaction times from the copolymerization of PA and PO performed in THF at RT with BDM as the initiator and the feed ratio of $[PA]_0/[PO]_0/[OH]_0/[tBuP_1]/[Et_3B]$ being 75/325/1/0.5/1.0, resulting in a gradient copolymer (G2 in Table 1). Conversions of PA and PO in Table 1 are calculated as a/(a+a') and d/(d+3e-3a'/4). S13

Figure S 5. ^{1}H NMR spectra of the aliquots withdrawn at different reaction times from the copolymerization of PA and PO performed in THF at RT with BDM as the initiator and the feed ratio of $[PA]_0/[PO]_0/[OH]_0/[tBuP_1]/[Et_3B]$ being 75/325/1/0.5/0.5, resulting in a gradient copolymer (G3 in Table 1). Conversions of PA and PO in Table 1 are calculated as a/(a+a') and d/(d+3e-3a'/4). S14

Figure S 6. DSC heating curves for PPO and P(POPA) homopolymers and PPO-co-P(POPA) copolymers, where the effect of gradient strength of the copolymer on the glass transition properties was studied. The P(POPA) homopolymer has a much higher glass transition temperature (T_g) compared to the PPO homopolymer (61 vs. −66 °C). The block copolymer and the gradient copolymers with the strongest and medium gradient strengths exhibit two glass transitions; the one belonging to the P(POPA) segments decreases and the one belonging to the PPG segments increases with decreasing gradient strength, indicating deteriorating microphase separation in this order. In contrast, the gradient copolymer with the weakest gradient has a single glass transition at −26 °C. S15

Figure S 7. SEC/MALS-RI chromatograms of copolymers recorded in $CHCl_3$ using Mixed D column. The pentagon label represents the molar mass of the copolymers, while triangle and square labels represent the molar masses of individual copolymer constituents (P(POPA) and PPO, respectively) as a function of elution volume. The solid and dashed lines represent the RI and 90° LS detector responses, respectively. S16

Figure S 8. LCCC-UV chromatograms for P(POPA) homopolymers, block (B) and gradient copolymers (G1, G2, G3) and LCCC-RI chromatogram of PPO-1 homopolymer together with molar mass as a function of elution volume obtained on PS-DVB reverse-stationary phase. PPO-2 is not shown because it did not elute from the column under critical conditions for P(POPA). Molar mass as a function of elution volume was determined by i) calculating the dn/dc value at each elution volume slice over copolymer peaks in the chromatograms according to Eq. 3 and using UV and RI concentration detectors (orange, light blue, light green, grey), and ii) by using the average dn/dc values of the copolymers (red, dark blue, light green, black) determined experimentally. In both cases, UV was used as a concentration detector. The two $M = f(V_{el})$ curves thus obtained for each copolymer agree well, confirming a narrow distribution in the chemical composition of the PPO-co-P(POPA) copolymers. It should be noted that the dn/dc values for the early eluting species of G1 and G2 and for the early eluting low intensity peaks of G3 and G4 cannot be calculated from a multidetection system, because the solvent peak partially overlaps with that of the copolymer peaks. For this reason, the $M = f(V_{el})$ curves in this case cannot be drawn over the entire peaks of G1 and G2 and over the low intensity peaks of G3 and G4. The composition of the mobile phase was THF : ACN = 15.6 : 84.4 vol% at 25 °C. S17

Figure S 9. SEC/MALS-RI chromatograms of copolymers recorded in $CHCl_3$ using successively coupled Mixed D and Mixed E SEC columns. The pentagon label represents the molar mass of the copolymers, while triangle and square labels represent the molar masses of individual copolymer constituents (P(POPA) and PPO, respectively) as a function of elution volume. The solid and dashed lines represent the RI and 90° LS detector responses, respectively. S18

Figure S 10. Schematic representation of fraction collection on PS-DVB reverse-stationary phase under critical conditions for P(POPA). The composition of the mobile phase was THF : ACN = 15.6 : 84.4 vol% at 25 °C. S19

Figure S 11. Schematic representation of fraction collection of G1 and G2 gradient copolymers on C18 reverse-stationary phase. The solvent gradient used ran from 0 – 65 % THF in ACN in 25 min. S20

List of Tables

Table S 1. Reaction conditions, conversion, molar mass averages, dispersity (Đ), specific refractive index increments (dn/dc), and extinction coefficients (ε) for PPO and P(POPA) homopolymers determined by SEC/UV-MALS-RI in $CHCl_3$. S8

Table S 2. Specific refractive index increments (dn/dc) and extinction coefficients (ε) of PPO and P(POPA) homopolymers in a solvent mixture THF : ACN = 15.6 : 84.4 vol. %. S8

Table S 3. Molar feed ratios, reaction times and conversions for gradient copolymers (G1, G2, G3) and block copolymer (B). S9

Table S 4. Weight-average molar masses (M_w) of the PPO-co-P(POPA) copolymers and their constituents as determined by SEC-MALS by calculating the *dn/dc* value at each elution volume slice over the peaks in chromatograms recorded in $CHCl_3$ and THF with refractive indices of 1.4459 and 1.4072, respectively. The M_w values of the copolymers determined on the basis of average *dn/dc* values of copolymers calculated according to Eq. 3 in Supp. Info., and experimentally determined *dn/dc* values assuming 100% mass recovery of the samples from the column are also given. In all cases, the tails on the low molar mass peak sides were excluded from the calculation. S9

Table S 5. Relative molar mass averages of spots in LCCC×SEC 2D-LC contour plots of copolymers as determined by calibration of SEC column with PS standards. S18

Table S 6. Chemical composition (POPA : PO) of the copolymer fractions determined by ^{1}H NMR and number-average molar masses of the G1 and G2 fractions and their P(POPA) and PPO constituents determined by SEC/UV-MALS-RI. The copolymer fractions were collected at the outlet of the C18 column under gLAC conditions according to the scheme shown in Figure S 11. ....S21

Experimental Section

Chemicals for synthesis

Tetrahydrofuran (THF; Guangzhou Chemical Reagent, AR) was successively dried over molecular sieve (4 Å) and calcium hydride (CaH_2) before distilled. Propylene oxide (PO; Aldrich, 99%) was stirred with sodium hydride (NaH) overnight at room temperature (RT), then vacuum-transferred into a storage flask. 1,4-Benzenedimethanol (BDM; Aladdin, 99%) was dried by azeotropic distillation of THF before use. Phthalic anhydride (PA; Aladdin, 99%) was first sublimated under vacuum, then dissolved in acetic anhydride and stirred at 80 °C overnight before recrystallized at RT. Acetic acid (AcOH; Aladdin, 99%), triethylborane (Et_3B, Aladdin, 1.0 M in THF) and *tert*-butylimino-tris(dimethylamino)phosphorane (tBuP_1; Aldrich, 97%) were used as received.

Synthesis of PPO homopolymers

Homopolymerization of PO is carried out at RT with the same catalyst composition as used for G1 (feed ratio of tBuP_1 and Et_3B is 0.5/1.5) and two different target degrees of polymerization, resulting in PPO homopolymers with two different molar masses (PPO-1 and PPO-2, Table S1).[62] A typical procedure for PPO-1 (Table S1) is as follows. BDM (6.9 mg, 0.05 mmol) was charged in a round-bottom reaction flask equipped with a Teflon stopcock and dissolved in purified THF, followed by slow removal of THF by vacuum-distillation. Then, PO (1.75 mL, 25.0 mmol), tBuP_1 (11.7 μL, 0.05 mmol), Et_3B (150 μL, 0.15 mmol), and THF (2.0 mL) were added in a glovebox ($[PO]_0$ = 6.4 M) to start polymerization. Small aliquots (ca. 0.05 mL each) were withdrawn at different time intervals, injected into $CDCl_3$ (0.6 mL, containing 1 drop of AcOH), and used for ^{1}H NMR analysis to determine the conversion of PO. After the reaction mixture was stirred for 96 h at RT, AcOH (1 mL) was added for quenching the polymerization. The solvent was removed by rotary evaporation, and the product (viscous liquid) was substantially dried in a vacuum oven at 45 °C. Conv.(PO) = 100%. Yield: 1.40 g (97%). Homopolymerization of PO for PPO-2 (Table S1) was performed according to similar procedure, except that a different amount of PO was added.

Synthesis of alternating copolymers of PA and PO (referred to as P(POPA) homopolymers)

Alternating copolymerization of PA and PO (free of PO self-propagation) is conducted with tBuP_1 as a single-component organocatalyst (0.5 equiv. of BDM hydroxyl group) at 60 °C also with two different degrees of polymerization targeted.[63] Full consumption of PA (PO is added in 0.5 eq. excess) and lack of PPO segments in the products, P(POPA)-1 and P(POPA)-2, are confirmed by ^{1}H NMR spectra. A typical procedure for P(POPA)-1 (Table S1) is as follows. PA (1.48 g, 10.0 mmol) and BDM (9.25 mg, 0.067 mmol) were charged in a round-bottom reaction flask equipped with a Teflon stopcock and dissolved in purified THF, followed by slow removal of THF by vacuum-distillation. Then, PO (1.05 mL, 15 mmol) and THF (2.0 mL) were added and the mixture was magnetically stirred at RT until PA dissolved completely. After that, tBuP_1 (15.7 μL, 0.067 mmol) was added in a glovebox ($[PA]_0$ = 3.3 M, $[PO]_0$ = 4.9 M) to start copolymerization. Small aliquots (ca. 0.05 mL each) were withdrawn at different time intervals, injected into $CDCl_3$ (0.6 mL, containing 1 drop of AcOH), and used for ^{1}H NMR analysis to determine the conversion of PA and PO. After the reaction mixture was stirred at 60 °C for 96 h, AcOH (1 mL) was added for quenching the polymerization. The crude product was diluted with THF, precipitated in methanol, collected, and dried in a vacuum to afford a white solid. Conv.(PA) = 100%. Yield: 2.01 g (98%).

Alternating copolymerization of PA and PO for P(POPA)-2 (Table S1) was performed according to similar procedure except that the different amounts of PA and PO were added.

Synthesis of PPO-co-P(POPA) copolymers

Synthesis of block copolymer from PA and PO (B in Table 1). PA (1.48 g, 10.0 mmol) and BDM (9.25 mg, 0.067 mmol) were charged in a round-bottom reaction flask equipped with a Teflon stopcock and dissolved in purified THF, followed by slow removal of THF by vacuum-distillation. Then PO (3.04 mL, 43.5 mmol) and THF (2.0 mL) were added and the mixture was magnetically stirred at RT until PA was completely dissolved. Then, *t*BuP$_1$ (15.7 µL, 0.067 mmol) and Et_3B (20.0 µL, 0.02 mmol) were added in a glovebox ($[PA]_0$ = 2.0 M, $[PO]_0$ = 8.6 M) to start copolymerization. Small aliquots (approximately 0.05 mL each) were withdrawn at different time intervals, injected into $CDCl_3$ (0.6 mL, containing 1 drop of AcOH), and used for ^{1}H NMR analysis to determine the conversion of PA and PO (Figure S1). After the reaction mixture was stirred for 96 h at RT, AcOH (1 mL) was added for quenching. The crude product was diluted with THF, precipitated in water, collected, and dried in vacuum to afford a white paste. Conv.(PA) = 100%, Conv.(PO) in the formation of PPO = 95%. Yield: 2.85 g (71%).

Synthesis of gradient copolymers from PA and PO (G1-3 in Table 1) was performed in a similar procedure, except that different amounts of Et_3B were added for each gradient copolymer (Table 1, Figures S2-4).

Calculation Procedure in SEC/UV-MALS-RI

The weight fraction of the UV-active component (in our case P(POPA)) at each peak slice in the SEC chromatogram is determined based on the fact that the concentration of the eluting PPO-*co*-P(POPA) copolymer species from the SEC column is the same whether they are monitored by the UV or RI concentration detectors (Eq. 1):

$$\frac{I_{\mathrm{RI}}}{K_{\mathrm{RI}}\left[\left(\frac{dn}{dc}\right)_{\mathrm{P(POPA)}}\cdot wt_{\mathrm{P(POPA)}}+\left(\frac{dn}{dc}\right)_{\mathrm{PPO}}\cdot\left(1-wt_{\mathrm{P(POPA)}}\right)\right]}=\frac{I_{\mathrm{UV}}}{K_{\mathrm{UV}}\left(\varepsilon_{\mathrm{P(POPA)}}\cdot wt_{\mathrm{P(POPA)}}\right)} \qquad \text{Eq. 1}$$

where I_{UV} and I_{dRI} represent the intensities of the UV and RI signals at each elution slice of the copolymer peak in the SEC chromatogram, K_{UV} and K_{RI} are the calibration constants for the UV and RI detectors, $\varepsilon_{\mathrm{P(POPA)}}$ is the extinction coefficient of the P(POPA) homopolymer, wt_{POPA} is the weight fraction of P(POPA) in the copolymer, while the $\left(\frac{dn}{dc}\right)_{\mathrm{PPO}}$ and $\left(\frac{dn}{dc}\right)_{\mathrm{POPA}}$ are the specific refractive index increments of the PPO and P(POPA) homopolymers, respectively.

The weight fraction of P(POPA) in PPO-*co*-P(POPA) at each slice of the peak in the SEC chromatogram of the copolymer can be derived from Eq. 1 and calculated according to equation 2:

$$wt_{\mathrm{P(POPA)}}=\frac{I_{\mathrm{UV}}K_{\mathrm{dRI}}\left(\frac{dn}{dc}\right)_{\mathrm{PPO}}}{I_{\mathrm{dRI}}K_{\mathrm{UV}}\varepsilon_{\mathrm{P(POPA)}}-I_{\mathrm{UV}}K_{\mathrm{dRI}}\left(\frac{dn}{dc}\right)_{\mathrm{P(POPA)}}+I_{\mathrm{UV}}K_{\mathrm{dRI}}\left(\frac{dn}{dc}\right)_{\mathrm{PPO}}} \qquad \text{Eq. 2}$$

By knowing the wt_{POPA}, the *dn/dc* value of the PPO-*co*-P(POPA) at each elution volume slice can be determined in the SEC chromatogram of the copolymer according to equation 3, which further allows to determine the concentration of the eluting species, the molar mass of the copolymer (Eq. 4) and its individual constituents (Eq. 5).

$$\left(\frac{dn}{dc}\right)_{\mathrm{copolymer}} = \left(\frac{\mathrm{dn}}{\mathrm{dc}}\right)_{\mathrm{PPO}} wt_{\mathrm{PPO}} + \left(\frac{\mathrm{dn}}{\mathrm{dc}}\right)_{\mathrm{P(POPA)}} wt_{\mathrm{P(POPA)}} \qquad \text{Eq. 3}$$

where $wt_{\mathrm{PPO}} = 1 - wt_{\mathrm{P(POPA)}}$

$$M_{\mathrm{PPO}-co-\mathrm{P(POPA)}} \propto \frac{I_{\mathrm{LS}}}{\left(\frac{dn}{dc}\right)_{\mathrm{PPO}-co-\mathrm{P(POPA)}} \cdot c_{\mathrm{PPO}-co-\mathrm{P(POPA)}}} \qquad \text{Eq. 4}$$

where I_{LS} is the intensity of the LS signal.

$$M_{\mathrm{P(POPA)\ in\ copolymer}} = \frac{(K_{\mathrm{RI}})^2}{K_{\mathrm{LS}} K_{\mathrm{UV}}} \frac{I_{\mathrm{LS}} I_{\mathrm{UV}}}{\varepsilon_{\mathrm{P(POPA)}} (I_{\mathrm{RI}})^2} \qquad \text{Eq. 5}$$

Fractionation of the copolymers by gLAC on the C18 reverse-stationary phase

The solutions of the copolymers in ACN with a typical concentration of 1.5 mg mL^{-1} were separated using the C18 reverse-stationary phase. The HPLC system, detectors, and on-line fraction collector were the same as described in the section: "Fractionation of copolymers by LCCC on the PS-DVB reverse-stationary phase," and the experiments were performed under the same conditions and with the same solvent gradient as described in the section gLAC for C18 column. Solvents were removed from the collected fractions by rotary evaporation and further dried in a vacuum oven at 50 °C. Such isolated fractions were analyzed by ^{1}H NMR and SEC/UV-MALS-RI to determine their chemical composition and molar mass characteristics.

Differential scanning calorimetry (DSC)

Measurements were performed in nitrogen atmosphere (flow rate: 20 mL/min) on a DSC 1 STAR^e system (Mettler Toledo; USA). The following heating/cooling program was used: heating the samples at a heating rate of 10 K/min from −80 to 150 °C; at 150 °C, the samples were tempered for 1 min and then cooled at a cooling rate of −200 K/min to −80 °C; finally, the samples were reheated to 150 °C at a heating rate of 20 K/min.

Results and Discussion

The (co)polymers investigated in this study were synthesized by ring-opening (co)polymerization using one- or two-component metal-free catalysts and a dihydroxy initiator (BDM) according to our previously reported methods.[61-63] THF is added in most cases to help with the dissolution of PA. For the synthesis of gradient and block copolymers, copolymerization is conducted at RT with the molar feed ratio of PA, PO, BDM hydroxyl

group (OH), and tBuP$_1$ kept at 75/325/1/0.5 and a varied amount of Et_3B (G1-3 and B in Table 1). It was previously found that in the copolymerization of PA and PO the relative activity of self-propagation of PO, generating PPO segments, elevates as more Et_3B is added.[61] Here, Et_3B is added in 1.5, 1.0, 0.5, and 0.15 equiv. (of BDM hydroxyl group) in the four copolymerization trials. PA conversion at which PO starts to self-propagate into PPO segments is found to be at least 0%, 55%, 74%, and 100% (Figures S1-S5), indicating the formation of gradient copolymers with the weakest, medium, and strongest gradient (G1-3), and a block copolymer (B), respectively. ^{1}H NMR spectra of the crude products also confirm full consumption of PO and PA for the synthesis of the above-mentioned copolymers (Figures S2-S5). Homopolymerization of PO is carried out at RT with the same catalyst composition as used for G1 (feed ratio of tBuP$_1$ and Et_3B is 0.5/1.5) and two different target degrees of polymerization, resulting in PPO homopolymers with two different molar masses (PPO-1 and PPO-2, Table S1).[62]. On the other hand, alternating copolymerization of PA and PO (free of PO self-propagation) is conducted with tBuP$_1$ as a single-component organocatalyst (0.5 equiv. of BDM hydroxyl group) at 60 °C also with two different degrees of polymerization targeted.[63] Full consumption of PA (PO is added in 0.5 eq. excess) and lack of PPO segments in the products, P(POPA)-1 and P(POPA)-2, are confirmed by ^{1}H NMR spectra.

Table S 1. Reaction conditions, conversion, molar mass averages, dispersity ($Đ$), specific refractive index increments (*dn/dc*), and extinction coefficients (ε) for PPO and P(POPA) homopolymers determined by SEC/UV-MALS-RI in $CHCl_3$.

Sample	$[PA]_0/[PO]_0/$ $[OH]_0/[t\mathrm{BuP}_1]/$ $[Et_3B]$	Time (h)	Conv. (%)*		$\bar{M}_n$ [kg/mol]	$\bar{M}_w$ [kg/mol]	$Đ$	dn/dc [mL/g]	ε [mL/(mg cm)]
			PA	PO in PPO					
PPO-1	-/250/1/0.5/1.5	96	-	100	17.3	17.9	1.04	0.025	0.047
PPO-2	-/500/1/0.5/1.5	96	-	100	33.5	35.5	1.06	0.026	0.039
P(POPA)-1	75/113/1/0.5/-	96	100	0	28.9	29.1	1.01	0.100	5.045
P(POPA)-2	150/2251/0.5/-	96	100	0	51.9	54.4	1.05	0.099	4.899

*Conversion of PA calculated by the integrals of the aromatic protons of PA and P(POPA) in ^{1}H NMR spectra, and conversion of PO in the formation of PPO calculated by the integrals of ether protons of PO and PPO. The dn/dc and ε were determined assuming 100% mass recovery of the homopolymers from the Mixed-D SEC column.

Table S 2. Specific refractive index increments (*dn/dc*) and extinction coefficients (ε) of PPO and P(POPA) homopolymers in a solvent mixture THF : ACN = 15.6 : 84.4 vol. %.

Sample	dn/dc [mL/g]	ε [mL/(mg cm)]
PPO-1	0.1110	0.001
P(POPA)-1	0.1815	5.294

Table S 3. Molar feed ratios, reaction times and conversions for gradient copolymers (G1, G2, G3) and block copolymer (B).

Sample	Molar feed ratio $[PA]_0/[PO]_0/[OH]_0/$ $[tBuP_1]/[Et_3B]$	Time (h)	Conv. (%)*	
			PA	PO in PPO
G1	75/325/1/0.5/1.5	72	>99	99
G2	75/325/1/0.5/1.0	72	100	100
G3	75/325/1/0.5/0.5	48	100	99
B	75/325/1/0.5/0.15	96	100	95

*Conversion of PA calculated by the integrals of the aromatic protons of PA and P(POPA) in ^{1}H NMR spectra, and conversion of PO in the formation of PPO calculated by the integrals of ether protons of PO and PPO, and aromatic protons of remaining PA (the amount of PO to be consumed in the formation of esters is subtracted in the calculation of this PO conversion; Figures S2-S5).

Table S 4. Weight-average molar masses (M_w) of the PPO-*co*-P(POPA) copolymers and their constituents as determined by SEC-MALS by calculating the *dn/dc* value at each elution volume slice[1] over the peaks in chromatograms recorded in $CHCl_3$ and THF with refractive indices of 1.4459 and 1.4072, respectively. The M_w values of the copolymers determined on the basis of average *dn/dc* values of copolymers calculated according to Eq. 3, and experimentally determined *dn/dc* values assuming 100% mass recovery of the samples from the column are also given. In all cases, the tails on the low molar mass peak sides were excluded from the calculation.

Sample	PPO[1] M_w / kgmol^{-1}		P(POPA)[1] M_w / kgmol^{-1}		PPO-*co*-P(POPA)[1] M_w / kgmol^{-1}		PPO-*co*-P(POPA) M_w / kgmol^{-1}			
							$CHCl_3$ with *dn/dc*		THF with *dn/dc*	
	$CHCl_3$	THF	$CHCl_3$	THF	$CHCl_3$	THF	calc.[2]	exp.[3]	calc.[2]	exp.[3]
G1	16.9	16.1	20.5	21.5	37.4	37.6	37.8	36.8	37.8	38.7
G2	17.1	16.7	25.5	25.7	42.6	42.4	42.4	41.1	42.6	43.4
G3	18.4	18.4	25.6	26.5	44.0	44.9	44.2	42.0	44.9	46.1
B	16.3	16.7	27.7	27.8	44.0	44.5	44.2	44.7	44.7	45.3

[1] Obtained by calculating the *dn/dc* value at each elution volume slice over the peaks in chromatograms.
[2] Obtained by average *dn/dc* of the copolymer calculated from the known average chemical composition and *dn/dc* values of the homopolymers according to Eq.3.
[3] Obtained by experimentally determined *dn/dc* from RI detector response in SEC chromatogram by taking into account 100% sample mass recovery from the column.

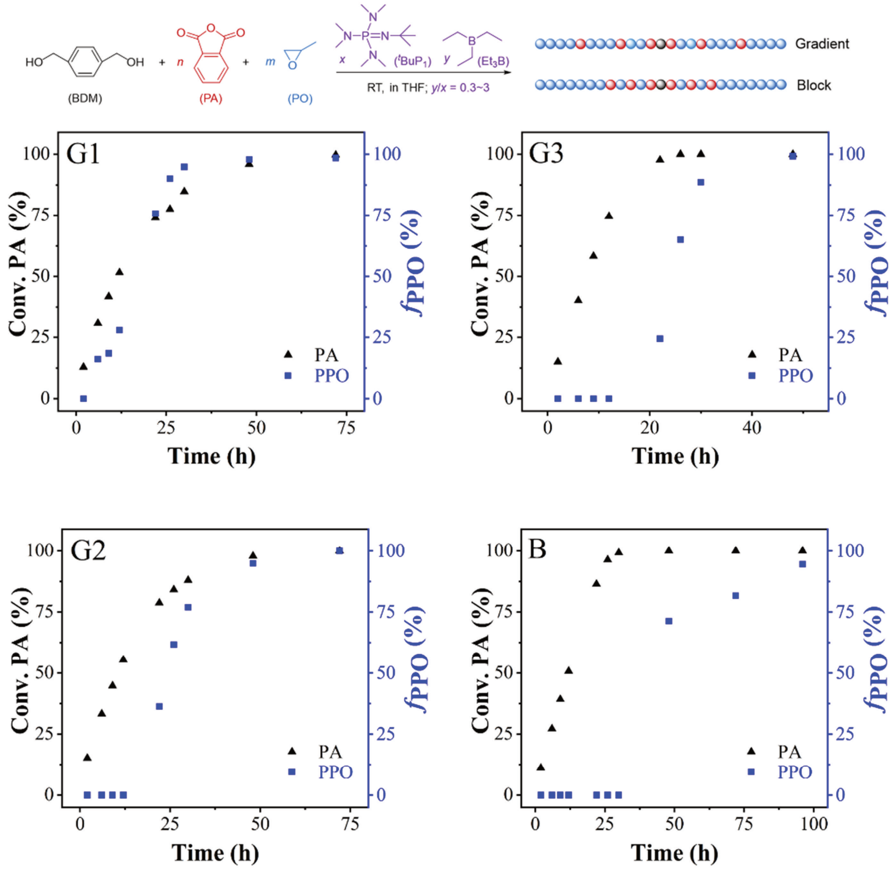

Figure S 1. Time dependence of the conversion of PA in the formation of POPA repeating units (or P(POPA) segments) (black triangles) and the conversion of PO in the formation of PPO segments (blue squares) during the copolymerization.

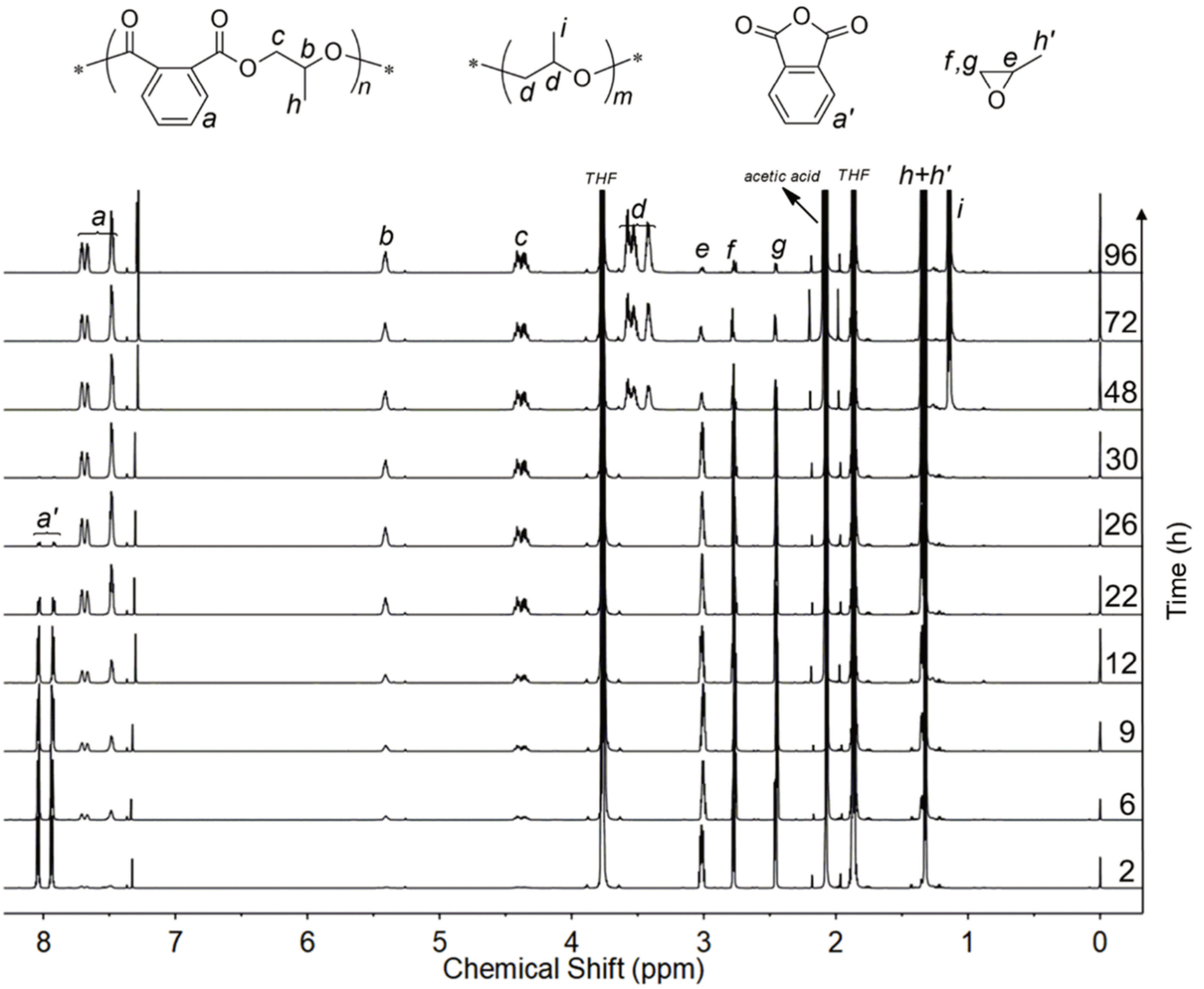

Figure S 2. ^{1}H NMR spectra of the aliquots withdrawn at different reaction times from the copolymerization of PA and PO performed in THF at RT with BDM as the initiator and the feed ratio of $[PA]_0/[PO]_0/[OH]_0/[tBuP_1]/[Et_3B]$ being 75/325/1/0.5/0.15, resulting in a block copolymer (B in Table 1). Conversions of PA and PO in Table 1 are calculated as a/(a+a') and d/(d+3e-3a'/4).

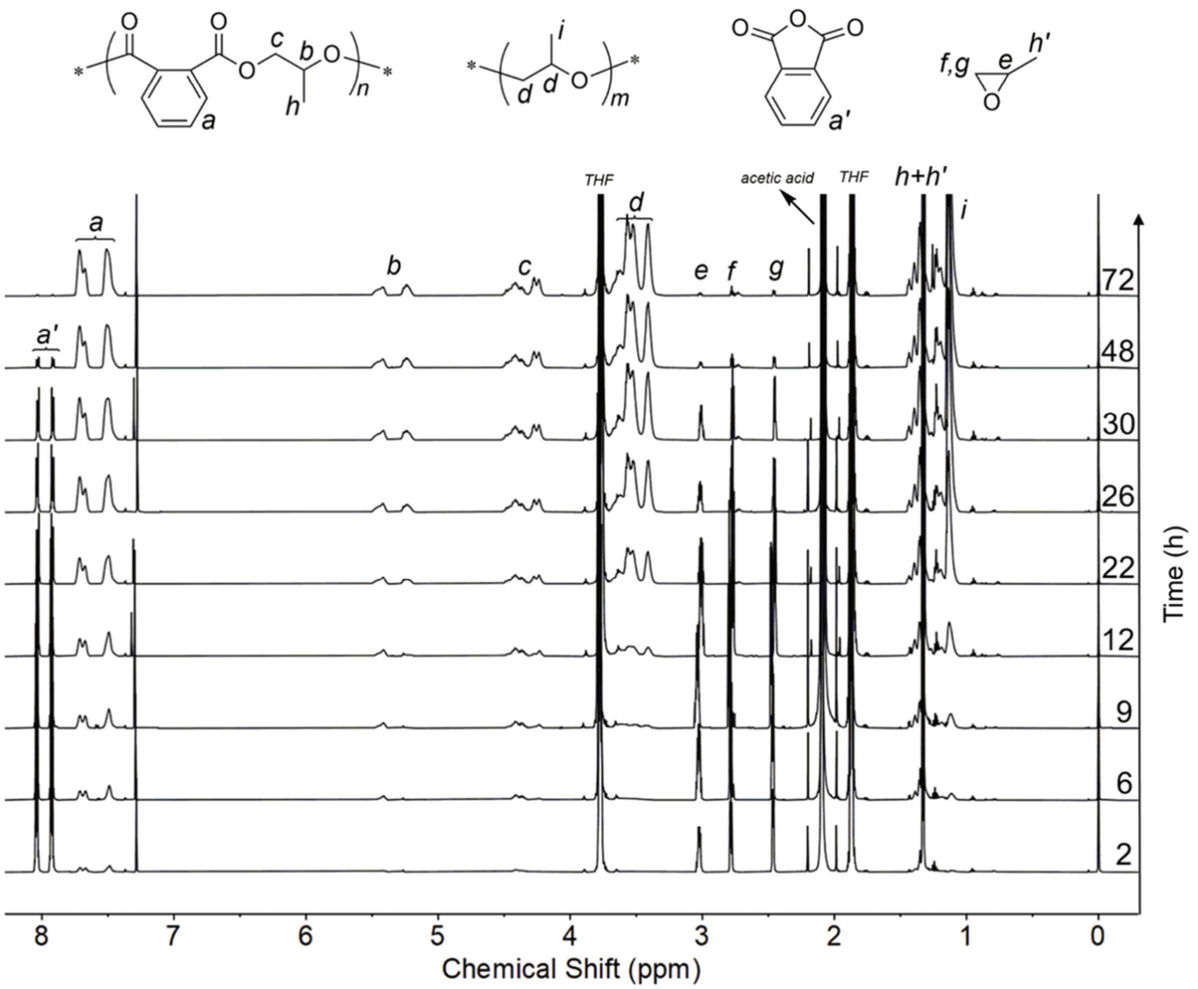

Figure S 3. ^{1}H NMR spectra of the aliquots withdrawn at different reaction times from the copolymerization of PA and PO performed in THF at RT with BDM as the initiator and the feed ratio of $[PA]_0/[PO]_0/[OH]_0/[tBuP_1]/[Et_3B]$ being 75/325/1/0.5/1.5, resulting in a gradient copolymer (G1 in Table 1). Conversions of PA and PO in Table 1 are calculated as a/(a+a') and d/(d+3e-3a'/4).

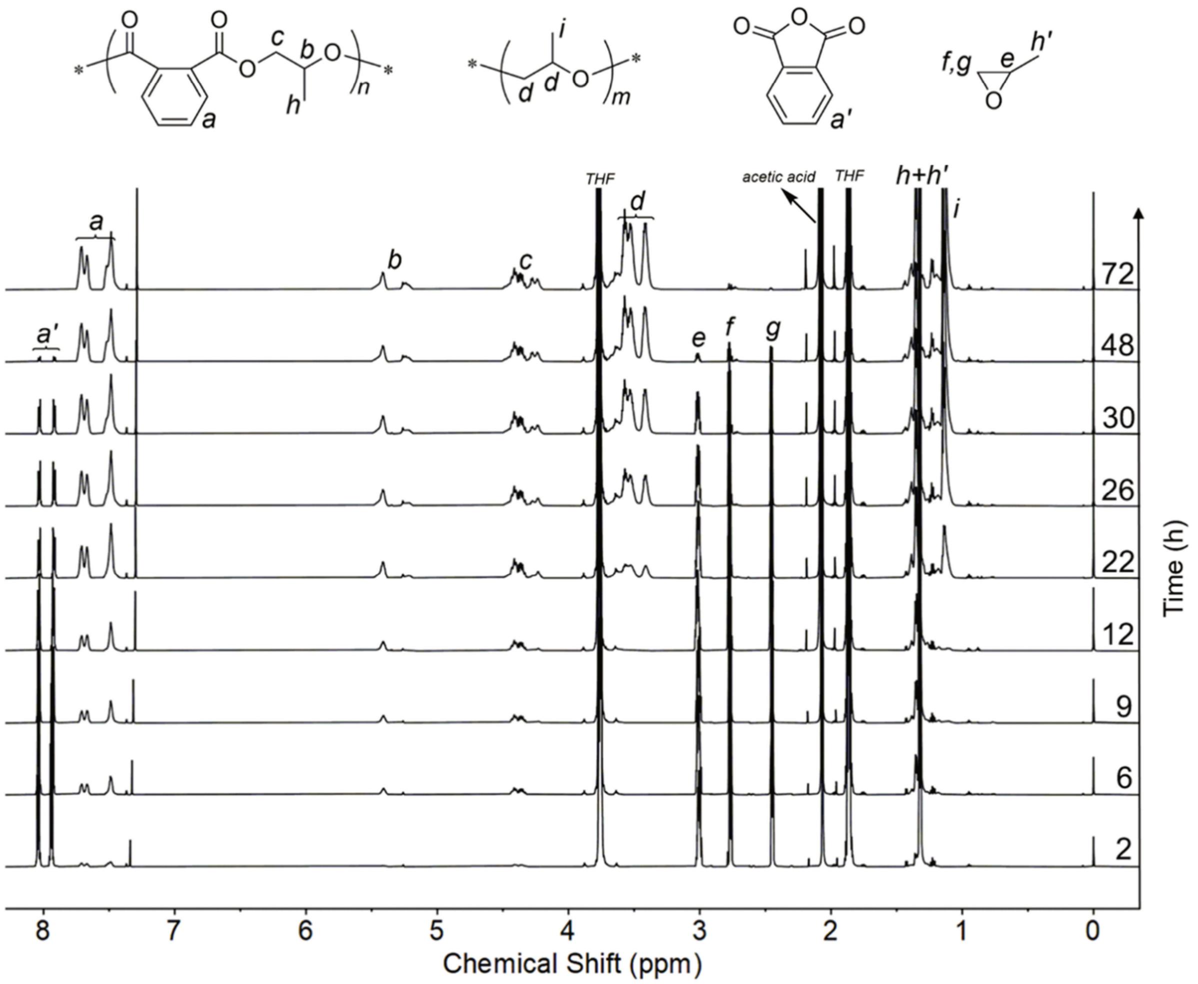

Figure S 4. ^{1}H NMR spectra of the aliquots withdrawn at different reaction times from the copolymerization of PA and PO performed in THF at RT with BDM as the initiator and the feed ratio of $[PA]_0/[PO]_0/[OH]_0/[t\text{BuP}_1]/[Et_3B]$ being 75/325/1/0.5/1.0, resulting in a gradient copolymer (G2 in Table 1). Conversions of PA and PO in Table 1 are calculated as a/(a+a') and d/(d+3e-3a'/4).

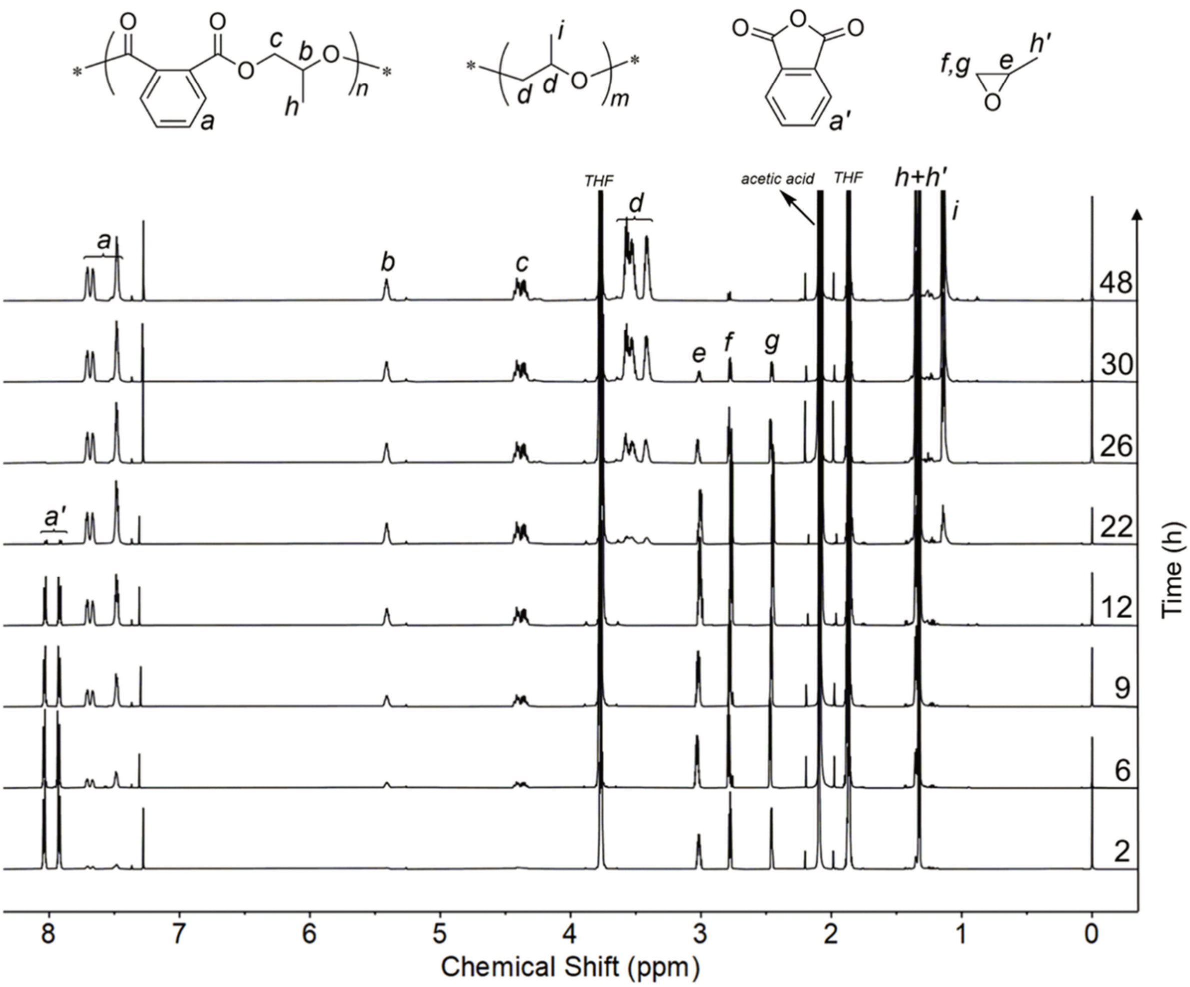

Figure S 5. ^{1}H NMR spectra of the aliquots withdrawn at different reaction times from the copolymerization of PA and PO performed in THF at RT with BDM as the initiator and the feed ratio of $[PA]_0/[PO]_0/[OH]_0/[t\text{BuP}_1]/[Et_3B]$ being 75/325/1/0.5/0.5, resulting in a gradient copolymer (G3 in Table 1). Conversions of PA and PO in Table 1 are calculated as a/(a+a') and d/(d+3e-3a'/4).

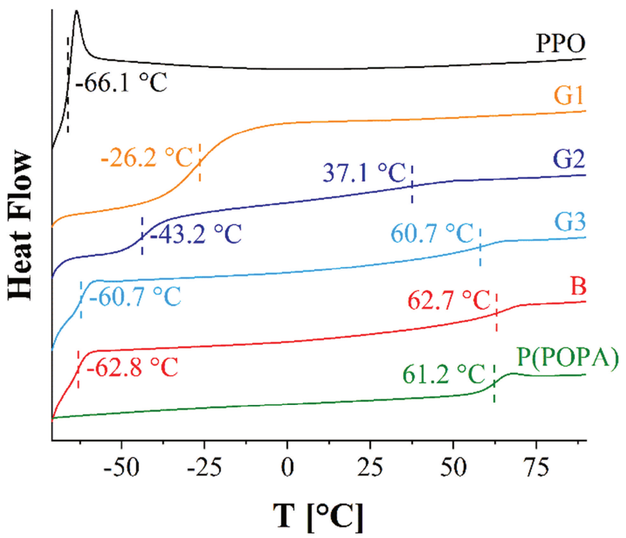

Figure S 6. DSC heating curves for PPO and P(POPA) homopolymers and PPO-*co*-P(POPA) copolymers, where the effect of gradient strength of the copolymer on the glass transition properties was studied. The P(POPA) homopolymer has a much higher glass transition temperature (T_g) compared to the PPO homopolymer (61 vs. −66 °C). The block copolymer and the gradient copolymers with the strongest and medium gradient strengths exhibit two glass transitions; the one belonging to the P(POPA) segments decreases and the one belonging to the PPG segments increases with decreasing gradient strength, indicating deteriorating microphase separation in this order. In contrast, the gradient copolymer with the weakest gradient has a single glass transition at −26 °C.

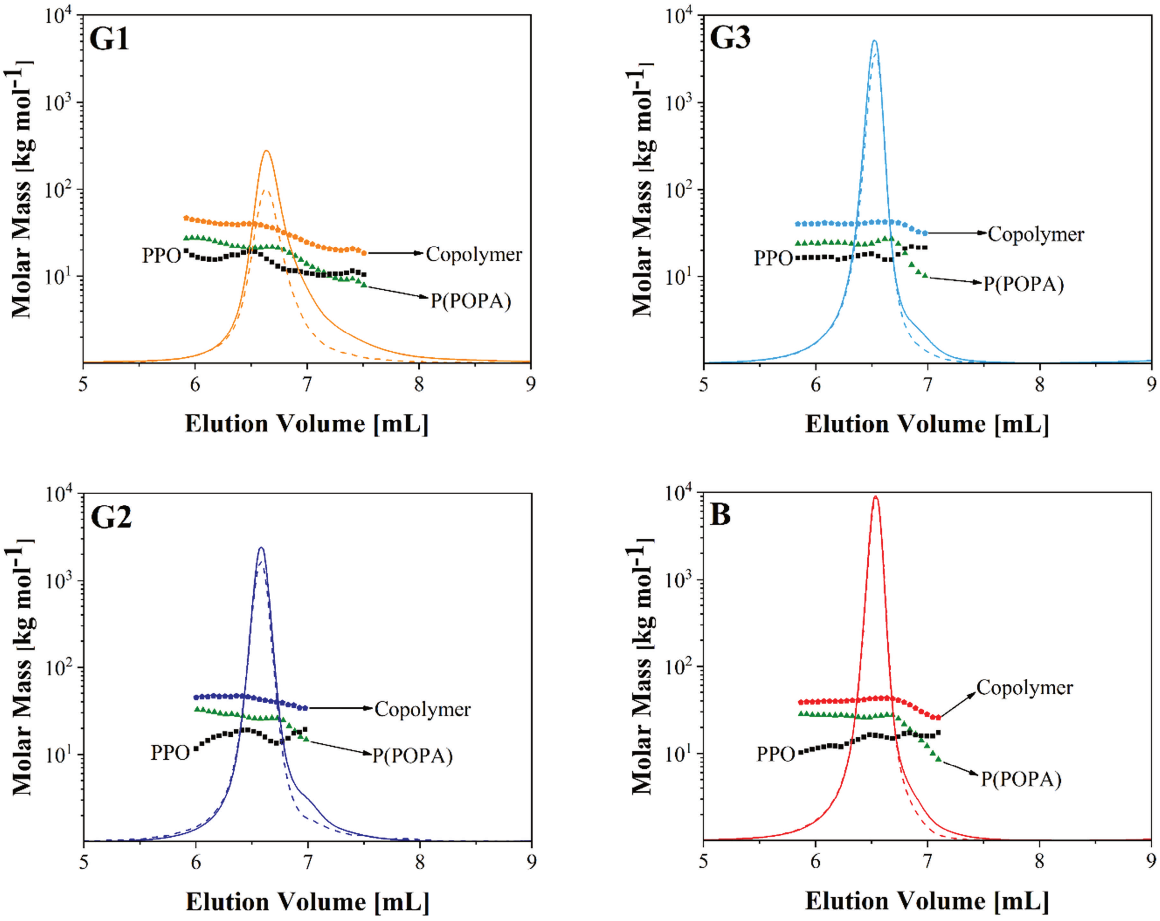

Figure S 7. SEC/MALS-RI chromatograms of copolymers recorded in $CHCl_3$ using Mixed D column. The pentagon label represents the molar mass of the copolymers, while triangle and square labels represent the molar masses of individual copolymer constituents (P(POPA) and PPO, respectively) as a function of elution volume. The solid and dashed lines represent the RI and 90° LS detector responses, respectively.

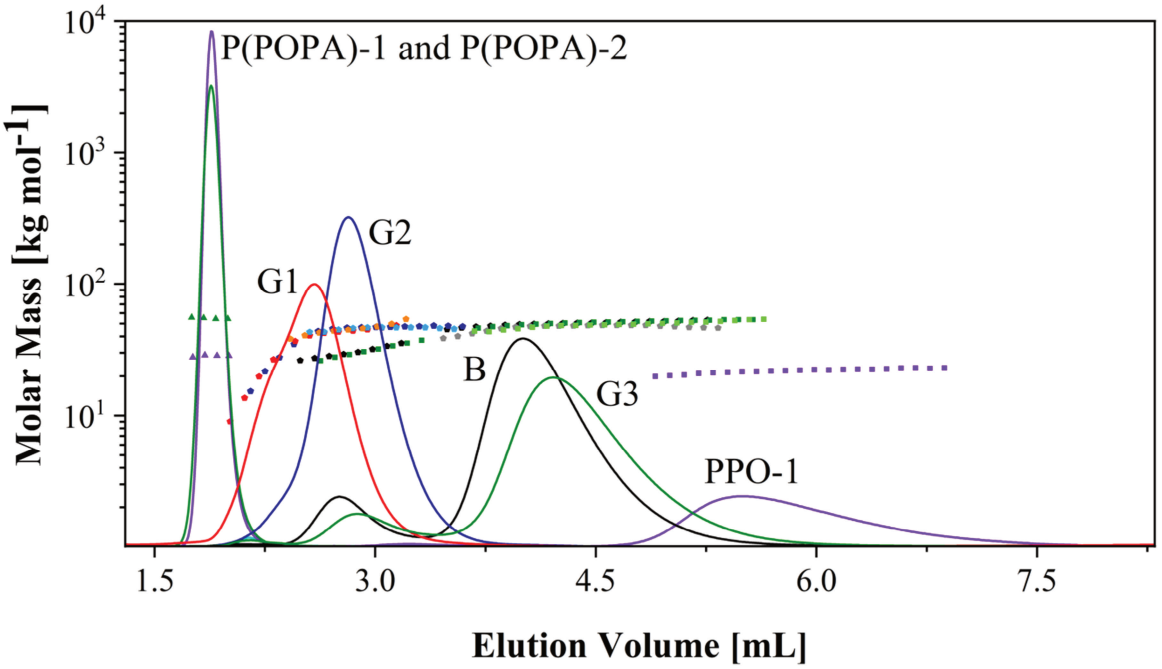

Figure S 8. LCCC-UV chromatograms for P(POPA) homopolymers, block (B) and gradient copolymers (G1, G2, G3) and LCCC-RI chromatogram of PPO-1 homopolymer together with molar mass as a function of elution volume obtained on PS-DVB reverse-stationary phase. PPO-2 is not shown because it did not elute from the column under critical conditions for P(POPA). Molar mass as a function of elution volume was determined by *i*) calculating the *dn*/*dc* value at each elution volume slice over copolymer peaks in the chromatograms according to Eq. 3 and using UV and RI concentration detectors (orange, light blue, light green, grey), and *ii*) by using the average *dn*/*dc* values of the copolymers (red, dark blue, light green, black) determined experimentally. In both cases, UV was used as a concentration detector. The two $M = f(V_{el})$ curves thus obtained for each copolymer agree well, confirming a narrow distribution in the chemical composition of the PPO-*co*-P(POPA) copolymers. It should be noted that the *dn*/*dc* values for the early eluting species of G1 and G2 and for the early eluting low intensity peaks of G3 and G4 cannot be calculated from a multidetection system, because the solvent peak partially overlaps with that of the copolymer peaks. For this reason, the $M = f(V_{el})$ curves in this case cannot be drawn over the entire peaks of G1 and G2 and over the low intensity peaks of G3 and G4. The composition of the mobile phase was THF : ACN = 15.6 : 84.4 vol% at 25 °C.

Table S 5. Relative molar mass averages of spots in LCCC×SEC 2D-LC contour plots of copolymers as determined by calibration of SEC column with PS standards.

Copolymer	Spot Intensity	$V_{el,appex}$ [mL]	$\overline{M}_n$ [kg·mol^{-1}]	$\overline{M}_w$ [kg·mol^{-1}]
G1	Low	1.98	15.7	17.5
	High	2.28	34.4	41.1
G2	Low	2.17	26.0	27.8
	High	2.50	35.7	41.1
G3	Low	2.41	25.8	27.5
	High	3.39	36.1	41.4
B	Low	2.33	26.5	28.9
	High	3.27	33.7	39.6

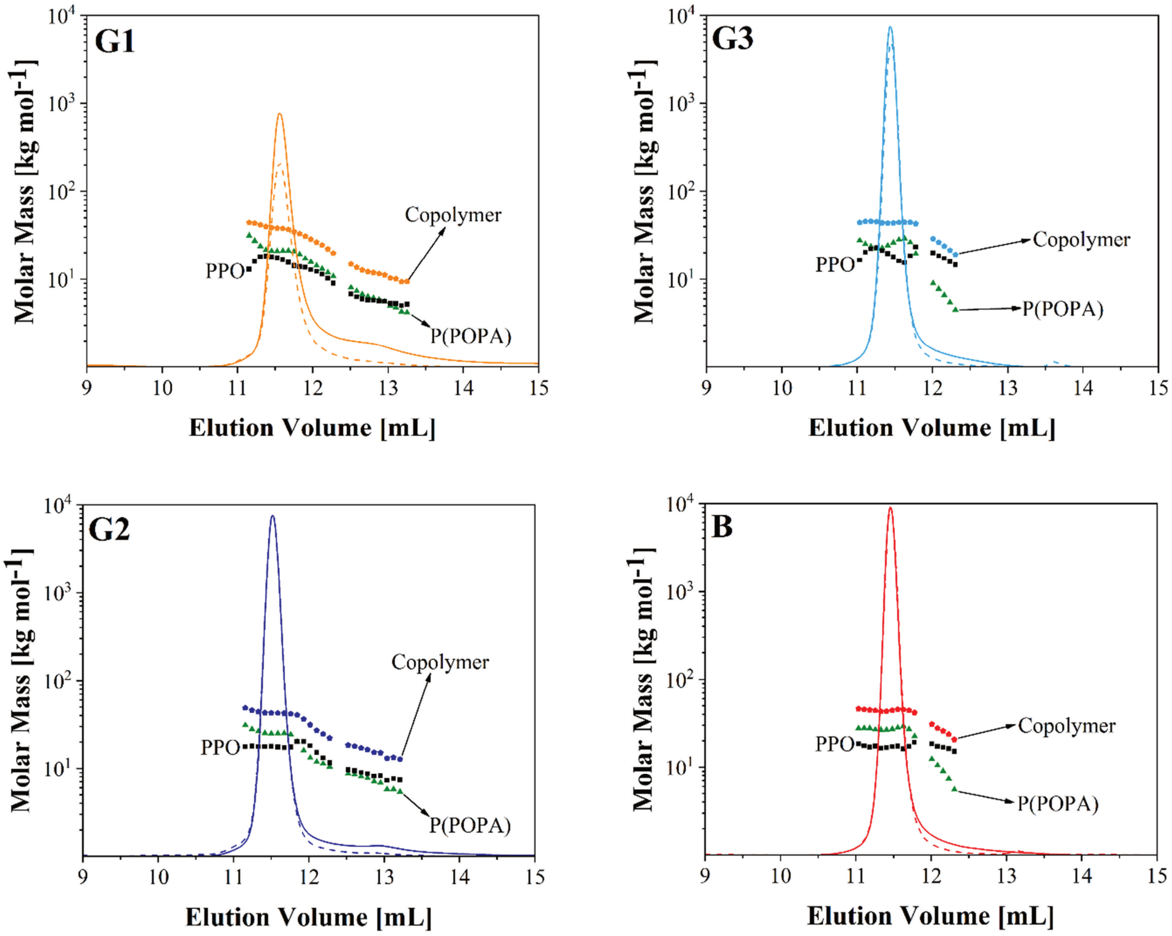

Figure S 9. SEC/MALS-RI chromatograms of copolymers recorded in $CHCl_3$ using successively coupled Mixed D and Mixed E SEC columns. The pentagon label represents the molar mass of the copolymers, while triangle and square labels represent the molar masses of individual copolymer constituents (P(POPA) and PPO, respectively) as a function of elution volume. The solid and dashed lines represent the RI and 90° LS detector responses, respectively.

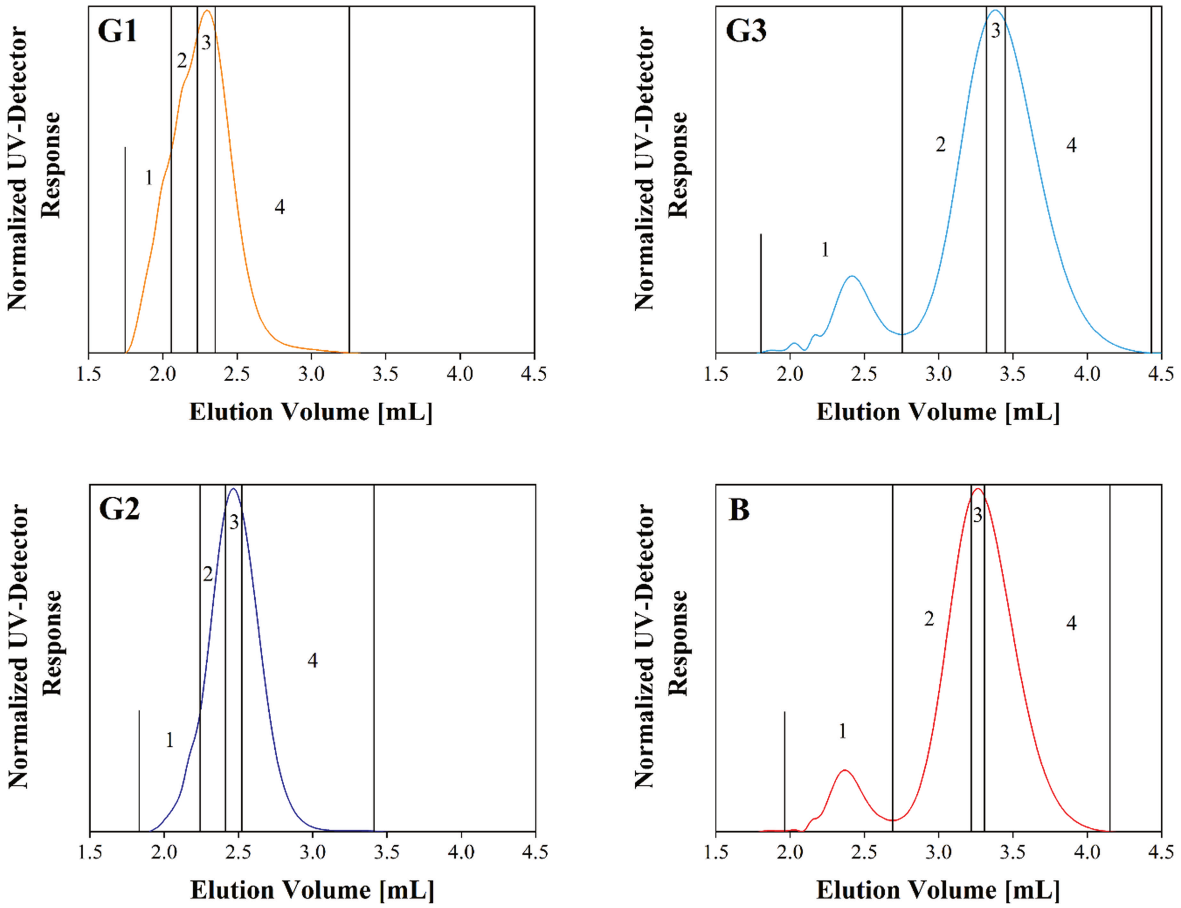

Figure S 10. Schematic representation of fraction collection on PS-DVB reverse-stationary phase under critical conditions for P(POPA). The composition of the mobile phase was THF : ACN = 15.6 : 84.4 vol% at 25 °C.

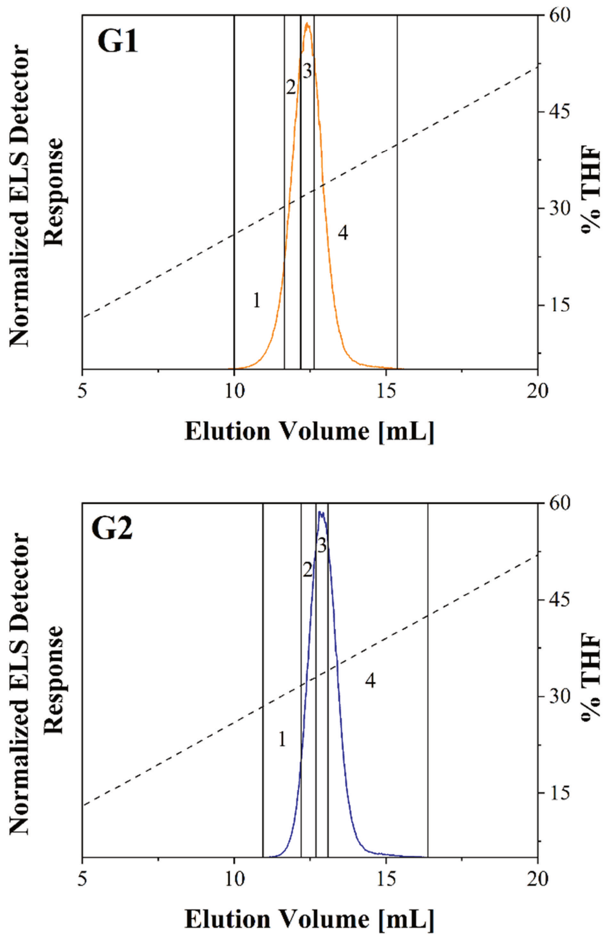

Figure S 11. Schematic representation of fraction collection of G1 and G2 gradient copolymers on C18 reverse-stationary phase. The solvent gradient used ran from 0 – 65 % THF in ACN in 25 min.

Table S 6. Chemical composition (POPA : PO) of the copolymer fractions determined by ^{1}H NMR and molar mass averages of the G1 and G2 fractions and their P(POPA) and PPO constituents determined by SEC/UV-MALS-RI. The copolymer fractions were collected at the outlet of the C18 column under gLAC conditions according to the scheme shown in Figure S 11.

Sample	Fraction Number	POPA : PO ^{1}H NMR [mol%]	Copolymer [kg/mol]		P(POPA) [kg/mol]		PPO [kg/mol]	
			$\overline{M}_n$	$\overline{M}_w$	$\overline{M}_n$	$\overline{M}_w$	$\overline{M}_n$	$\overline{M}_w$
G1	1	27.6 : 72.4	15.0	19.5	10.3	11.1	4.9	8.4
	2	27.6 : 72.4	29.6	30.3	17.9	18.4	11.6	11.9
	3	25.6 : 74.4	34.4	35.1	19.5	19.6	14.9	15.5
	4	23.5 : 76.5	26.4	27.4	14.8	15.1	11.6	12.3
G2	1	32.3 : 67.7	21.8	28.3	12.7	17.8	6.9	10.5
	2	32.2 : 67.8	36.6	37.0	25.2	25.2	11.2	12.7
	3	30.0 : 70.0	39.3	39.4	24.3	24.4	14.9	14.9
	4	25.1 : 74.9	34.2	34.4	21.3	21.4	12.8	13.2
